# Dual-adaptive imputation graph neural network for knowledge-aware recommendation

**DOI:** 10.1038/s41598-026-57267-x

**Published:** 2026-06-13

**Authors:** Zhenge Huo, Huanhuan Liu, Xinglong Wu, Chenxing Xia, Bin Ge, Yu Zhang

**Affiliations:** 1 School of Computer Science and Engineering, Anhui University of Science and Technology, Huainan, 232001 China; 2 State Key Laboratory of Digital Intelligent Technology for Unmanned Coal Mining, Anhui University of Science and Technology, Huainan, 232001 China

**Keywords:** Graph neural networks, Knowledge graphs, Recommender systems, Feature fusion, Engineering, Mathematics and computing

## Abstract

Recommender systems play a vital role in enhancing user experience by efficiently delivering personalized and relevant content. While knowledge graph-based recommender systems effectively alleviate the data sparsity and cold-start challenges of traditional approaches, they still suffer from two major limitations: (1) insufficient utilization of the user-item interaction matrix and (2) suboptimal integration of heterogeneous knowledge graph signals with collaborative information. In this work, we propose DAIGNN (Dual-Adaptive Imputation Graph Neural Network), a novel recommendation framework designed to overcome these limitations through three key innovations. First, we introduce a similarity-driven imputation mechanism that constructs an Imputation Graph using pseudo-ratings, thereby enhancing graph connectivity and significantly reducing data sparsity. Second, we incorporate multiple auxiliary information sources on both the user and item sides, enabling DAIGNN to capture richer contextual and relational semantics beyond conventional user-item interactions. Third, we develop a dual-adaptive feature fusion mechanism that learns optimal fusion weights to dynamically integrate heterogeneous information from multiple graph sources. Extensive experiments conducted on four real-world datasets demonstrate the superior effectiveness of DAIGNN. On average, it achieves a 3.1% improvement in AUC and a 2.0% improvement in F1-score over state-of-the-art baselines, confirming its robustness across diverse settings.

## Introduction

Knowledge graph (KG)-based recommender systems enrich collaborative filtering with structured semantic relations among entities, alleviating the data sparsity and cold-start limitations of purely interaction-driven approaches^1–3^. Beyond recommendation, graph-based learning has been successfully applied across a broad spectrum of domains^4–7^, including computer vision^8,9^ and intelligent education^10^, underscoring the generality of graph-structured modeling. Within KG-aware recommendation, recent work has advanced both representation learning (e.g. GNN-based KG propagation^11–13^) and multi-source information fusion (e.g. contrastive and attention-based strategies^14–16^), while hierarchical attention has been applied to context-aware modeling of item attributes and user intentions in sequential recommendation^17–20^. Despite this progress, two critical design gaps remain.

**Fig. 1:**
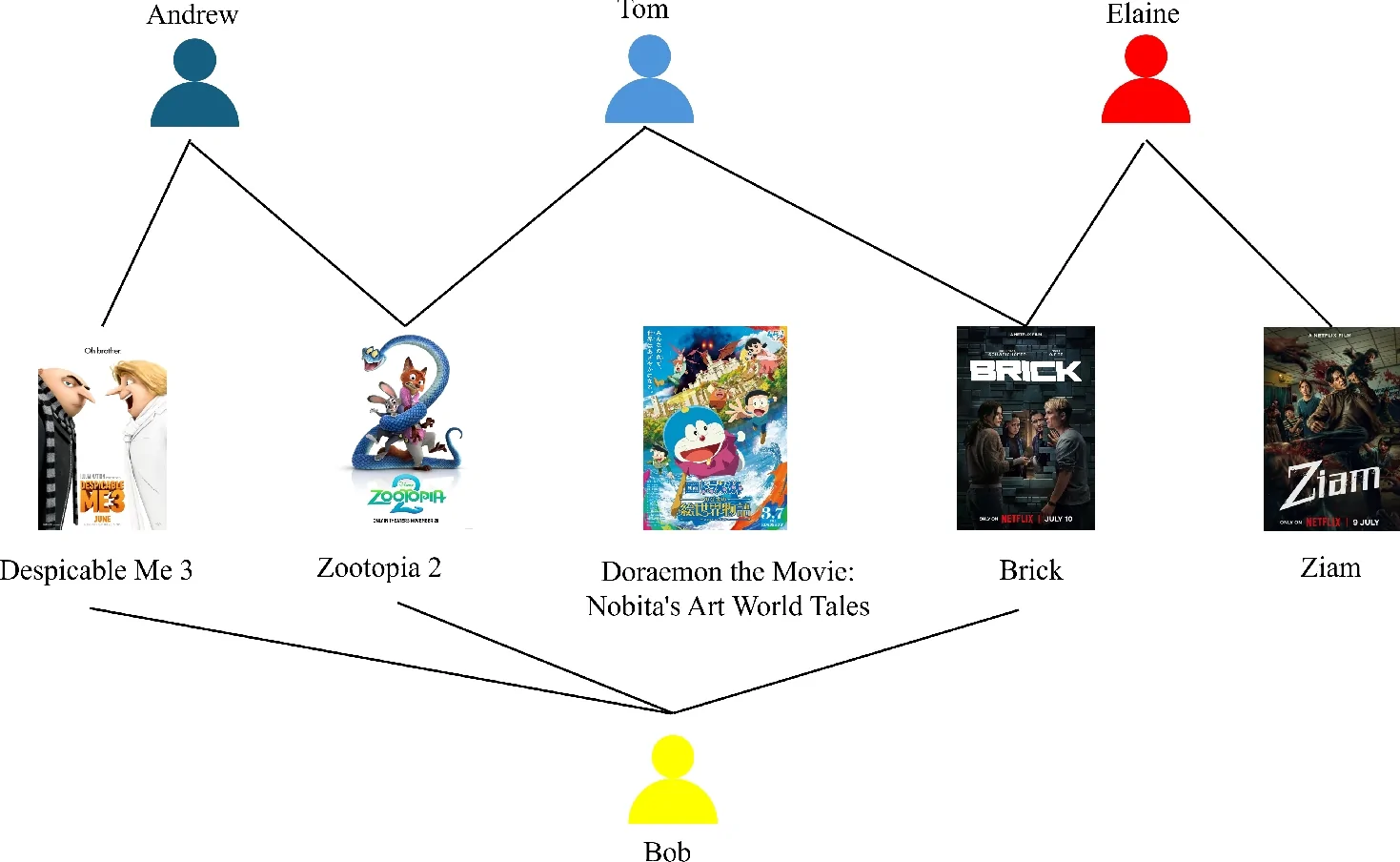
Illustrative user-movie interaction showing how indirect behavioral similarity reveals meaningful user-item relevance. Andrew’s similarity to Bob suggests that “Brick” should be considered relevant despite the absence of a direct interaction, highlighting the cost of treating all unobserved items as negatives.

1. *The structural sparsity of the user-item interaction graph is not resolved at the graph level.* Existing models learn user representations solely from observed interactions, treating all unobserved items as negatives. This conflates lack of exposure with lack of interest, introducing systematic exposure bias^21^. Figure 1 illustrates the issue: Andrew interacted only with two cartoon movies, yet his behavioral similarity to Bob implies that the movie “Brick” is also relevant. Discarding such unobserved yet plausible items forfeits informative collaborative signals. Contrastive-learning methods^14,16^ improve embedding quality through self-supervised objectives, but they do not add new edges to the interaction graph; the sparse topology therefore remains unchanged at the structural level.
2. *Heterogeneous feature fusion remains non-adaptive across information sources.* Most KG-based models integrate collaborative and knowledge-graph signals via concatenation or fixed linear weighting. This strategy assumes all feature spaces share compatible statistics, an assumption that rarely holds when signals originate from structurally distinct graphs. Without explicit cross-source modulation, dominant features overshadow weaker yet informative ones, and incompatible embeddings introduce noise. Existing attention mechanisms^11,22,23^ weight neighbors within a single graph; they cannot resolve conflicts *across* heterogeneous information sources.

To address these challenges, we propose *Dual-Adaptive Imputation Graph Neural Network (DAIGNN)*. DAIGNN introduces a similarity-driven imputation mechanism that infers pseudo-ratings from behaviorally similar users and selectively adds high-confidence edges to the interaction graph, thereby densifying its structure with a semantically grounded third information channel that is absent from all existing KG-based recommenders. Concurrently, a cross-source adaptive fusion module learns global importance scores for heterogeneous embedding sources on both the user side and the item side, dynamically reconciling signals from structurally distinct graphs. The main contributions are:

- *Imputation graph construction.* We infer pseudo-ratings via top-*k* user similarity (Pearson or cosine, adaptively selected) and retain only edges exceeding a confidence threshold, providing an additional imputation-neighbor embedding that captures latent collaborative signals unexpressed in the observed data.
- *Dual-adaptive feature fusion.* We design an asymmetric attention mechanism that computes cross-source importance weights for three user-side embeddings (user, user-relation interaction, imputation neighbor) and four item-side embeddings (fused user, entity, relation, item-user interaction). Unlike intra-graph attention, this mechanism explicitly resolves conflicts across heterogeneous graphs.
- *Empirical validation.* Experiments on four real-world datasets demonstrate that DAIGNN consistently outperforms state-of-the-art baselines, achieving average improvements of 3.1% in AUC and 2.0% in F1-score. Ablation and attention-weight analyses confirm the complementary contributions of the imputation graph and adaptive fusion.

## Related work

KG-based recommendation enriches collaborative filtering with structured semantic relations, alleviating the data sparsity and cold-start limitations of purely interaction-driven approaches. Existing methods can be broadly categorized into embedding-based and GNN-based paradigms. Embedding-based methods constitute the first generation of KG-aware recommenders, while GNNs have become the dominant paradigm by addressing the depth limitation through recursive neighborhood aggregation. We organize GNN-based approaches into three thematic groups–knowledge graph convolution, multi-channel and contrastive representation learning, and attention-based relational reasoning–and review each with an emphasis on the design principles they share and the structural gaps that motivate our work.

### Embedding-based methods

Embedding-based methods constitute the first generation of KG-aware recommenders. They rely on knowledge graph embedding (KGE) techniques to project entities and relations into low-dimensional vector spaces, which are then integrated into collaborative filtering models. Two complementary design strategies have been explored within this paradigm.

The first strategy fuses heterogeneous item-side signals through KGE techniques. Collective Knowledge Embedding (CKE)^24^ exemplifies this approach, integrating structural knowledge, textual descriptions, and visual features into unified item embeddings. Knowledge-aware Transferable User Preference (KTUP)^25^ extends multi-source fusion through multi-task learning that jointly optimizes user-item interactions and KG relational triples, enabling structured semantic knowledge to directly inform user preference modeling. The second strategy adapts translational KGE models originally developed for KG completion. TransR^26^ and TransH^27^ project entities and relations into distinct vector spaces to capture diverse relational semantics, while RotatE^28^ models complex relation patterns–including symmetry, inversion, and composition–through rotational transformations in complex vector space, enriching entity representations beyond simple vector embeddings.

Despite their foundational contributions, embedding-based methods share two structural limitations that constrain their recommendation performance. First, their reliance on first-order neighborhood information prevents them from capturing multi-hop semantic dependencies. A user’s preference may be mediated through paths spanning several relational hops (e.g., user *→* movie *→* director *→* genre), and first-order methods can only exploit immediate connections. Second, many of these methods optimize objectives designed for KG completion (predicting missing triples) rather than recommendation (predicting user-item interactions). This objective mismatch relegates collaborative signals–the most direct indicators of user preference–to an auxiliary role, limiting their influence on the learned representations. Beyond KG embedding techniques, reinforcement learning-based multi-hop reasoning has achieved strong results on few-shot KG completion^29^, and cross-domain preference transfer has been explored to mitigate cold-start in location-based recommendation^30^. However, these approaches target KG reasoning and cross-domain adaptation respectively, rather than end-to-end KG-aware recommendation with enriched graph structure. These limitations motivate the adoption of GNN architectures that can recursively aggregate information across multi-hop neighborhoods and jointly optimize for recommendation.

### Knowledge graph convolution

KGCN^11^ pioneered the use of graph convolution for KG-based recommendation by constructing user-specific weighted subgraphs that tailor knowledge propagation to individual preferences. By replacing uniform neighborhood aggregation with preference-dependent weighting, KGCN demonstrated that personalization at the propagation level yields substantial gains over embedding-based methods. KGNN-LS^31^ improves upon this framework by introducing label smoothness regularization, which enforces the inductive bias that adjacent items in the KG are likely to share similar user relevance scores, thereby regularizing edge weights during propagation. Both models, however, share a fundamental architectural constraint: their attention mechanisms operate exclusively within the knowledge graph, weighting individual KG neighbors without any capacity to compare, reconcile, or dynamically modulate information originating from structurally distinct graphs (e.g., the user-item interaction graph versus the knowledge graph). Since these graphs carry fundamentally different statistical properties–interaction signals are sparse and behaviorally grounded, whereas KG relations are dense and semantically structured–treating intra-graph attention as a proxy for cross-source fusion introduces an implicit assumption that all useful information resides within a single graph structure.

### Multi-channel and contrastive representation learning

Recognizing that collaborative signals and knowledge signals carry complementary information, several methods have adopted multi-channel architectures. CKAN^32^ employs a dual-channel GCN^33^ design that applies distinct aggregation mechanisms on the user-item interaction graph and the knowledge graph, successfully separating the two signal types. However, it aggregates multi-layer representations from the two channels through fixed-structure operators (e.g., sum, pooling, or concatenation followed by a linear transformation). This static fusion strategy implicitly assumes that all information sources contribute equally across all samples–an assumption violated when, for instance, collaborative signals are strong for popular items but weak for long-tail items with few interactions. KRNN^34^ addresses over-smoothing in deeper GNN layers through a random dropout strategy that generates multiple perturbed entity feature matrices as data augmentations, with consistency regularization optimizing predictions across different augmentation views. While this improves robustness, KRNN operates on a single KG-derived graph and does not provide a mechanism for dynamically weighting information from structurally distinct sources. Outside the KG-aware paradigm, GNN-based collaborative filtering models have explored adaptive edge weighting and high-order spatial connectivity mining to mitigate over-smoothing in interaction graphs^35^, though these methods do not exploit the rich relational semantics of knowledge graphs.

In a complementary direction, KGIC^14^ and KGSL^16^ integrate contrastive learning through self-supervised objectives. KGIC performs layer-wise interactive contrastive learning between the CF and KG parts within local and non-local KG-derived graphs, maximizing mutual information across different views of the same node. KGSL constructs a semantic item-similarity graph from KG relations and generates contrastive views from both the interaction graph and this similarity graph. These methods improve representation quality without additional labels, but they share a critical limitation with respect to data sparsity: neither introduces new edges into the user-item interaction graph itself. KGIC expands which KG facts are considered through non-local graphs, and KGSL adds an item-similarity view, but the user-item interaction topology–the primary conduit for collaborative signals–remains unchanged. Contrastive objectives can sharpen existing representations but cannot recover preference signals for user-item pairs that are structurally disconnected. This distinction–improving representations given a fixed interaction graph versus enriching the interaction structure itself–is fundamental, and existing multi-channel and contrastive methods address only the former. More recently, graph rewiring techniques have been applied to collaborative filtering, pruning low-contribution interaction edges to construct a sparser but more informative propagation topology^36^. This represents a complementary structural modification strategy–edge removal rather than edge addition–that further highlights the value of directly manipulating interaction graph topology to improve recommendation performance.

### Attention-based relational reasoning

Several methods focus on designing more expressive attention mechanisms within individual graphs. NKGAT^22^ introduces Neighbor-Augmented Attention that assigns differentiated weights to nodes at the same propagation hop, enabling finer-grained neighbor discrimination than uniform-hop aggregation. KGARA^23^ develops adaptive relational attention that modulates the contribution of different relation types, integrating relation-aware receptive fields. MI-KGNN^37^ employs dual-attention to jointly model structural and semantic dependencies through personalized KGs. KEGNN^38^ targets explainable recommendation by constructing a user behavior graph that integrates KG semantics with interaction edges, then applying GNN-based reasoning over this graph for both rating prediction and natural-language explanation generation via an RNN-based decoder. SMCR^39^ extends GNN reasoning to conversational recommendation through multi-hop attention that encodes both KG structure and long-term dialogue dependencies, using sparse edge identification to reduce computational complexity on noisy conversational KGs. RFA-KG^40^ introduces relation-fused attention with cross-compression networks and dynamic gate control, strengthening entity-relation interactions through contrastive learning. Collectively, these methods progressively improve intra-graph attention.

Beyond KG-based architectures, attention mechanisms have been extensively explored in sequential and context-aware recommendation. DIARec^17^ captures dynamic user intentions through hierarchical item-attribute relations, HAN^18^ and CARA^20^ employ hierarchical attention with positional encoding for temporal dependency modeling, and GCORec^19^ integrates group-wise attention with recurrent networks for collaborative filtering. In cross-domain sequential recommendation, multimodal fusion and large language models have been leveraged to enrich item representations: IFCDSR^41^ incorporates image features via CLIP, LLM-EMF^42^ enhances textual data through LLMs, and TEMA-LLM^43^ generates semantic tags with LLM-based multi-attention. While effective for context modeling and cross-domain transfer, these methods operate outside the KG paradigm and do not address the structural sparsity or cross-source fusion challenges that motivate DAIGNN.

However, across all three groups, two structural limitations persist. First, attention mechanisms remain confined to intra-graph weighting; when a model draws information from multiple graphs with distinct statistical properties, it lacks a principled mechanism for learning cross-source importance weights. Second, the sparsity of the user-item interaction graph remains unaddressed at the topological level–observed edges are the only message-passing pathways regardless of whether attention is personalized, multi-channel, or contrastive. DAIGNN addresses both limitations through its similarity-driven imputation graph and cross-source adaptive fusion mechanism.

## Problem formulation

This section defines the notation and the two primary data sources–the user-item interaction matrix and the knowledge graph–used throughout this paper. Table 1 summarizes the key symbols for ease of reference.

**Table 1:** Summary of key notations.

Symbol	Description
$\mathscr {U}$, $\mathscr {I}$	User set, item set
*M*, *P*	Number of users, number of items
$\mathscr {Y} \in \{0,1\}^{M \times P}$	User-item interaction matrix
$\mathscr {G}$, $\mathscr {E}$, $\mathscr {R}$	Knowledge graph, entity set, relation set
(*h*, *r*, *t*)	A KG triplet (head, relation, tail)
*d*	Embedding dimension
*b*	Mini-batch size
*K*	Number of sampled neighbors (KG or imputation)
$\textrm{sim}(u,v)$	Similarity between users *u* and *v* (Pearson or cosine)
$\tilde{y}_{ui}$	Pseudo-rating of user *u* for item *i*
*τ*	Imputation edge confidence threshold
$w_{ui}$	Normalized confidence weight for imputation edge (*u*, *i*)

### User-item interaction data

In a standard recommendation scenario, we define the user set $\mathscr {U}=\{u_1,u_2,\ldots ,u_M\}$ and the item set $\mathscr {I}=\{i_1,i_2,\ldots ,i_P\}$, where *M* denotes the total number of users and *P* represents the total number of items. The interaction matrix $\mathscr {Y}\in \{0,1\}^{M\times P}$ is established from implicit user feedback. Specifically, $y_{ui}=1$ indicates that user *u* has interacted with item *i* (e.g., clicked, viewed, or purchased), whereas $y_{ui}=0$ implies that no interaction has been recorded.

### Knowledge graph

Item-related semantic information–such as product attributes and categorical relations–is organized as a heterogeneous structured graph that goes beyond user-item interactions. We denote the KG as $\mathscr {G}=\{(h,r,t)\mid h,t\in \mathscr {E},\; r\in \mathscr {R}\}$, where each triplet (*h*, *r*, *t*) represents a directed relation *r* from head entity *h* to tail entity *t*. The sets $\mathscr {E}$ and $\mathscr {R}$ contain all entities and relation types, respectively. In practice, each item $i\in \mathscr {I}$ is mapped to a corresponding entity $e\in \mathscr {E}$ in the KG, allowing the knowledge graph to augment interaction data with high-level semantic and relational context.

### Task definition

Given the interaction matrix $\mathscr {Y}$ and the KG $\mathscr {G}$, our goal is to learn a predictive function $\hat{y}_{ui}= f(u,i\mid \mathscr {Y},\mathscr {G},\Theta )$ that estimates the probability that user *u* will interact with item *i*, where *Θ* denotes all learnable model parameters.

## Proposed method

**Fig. 2:**
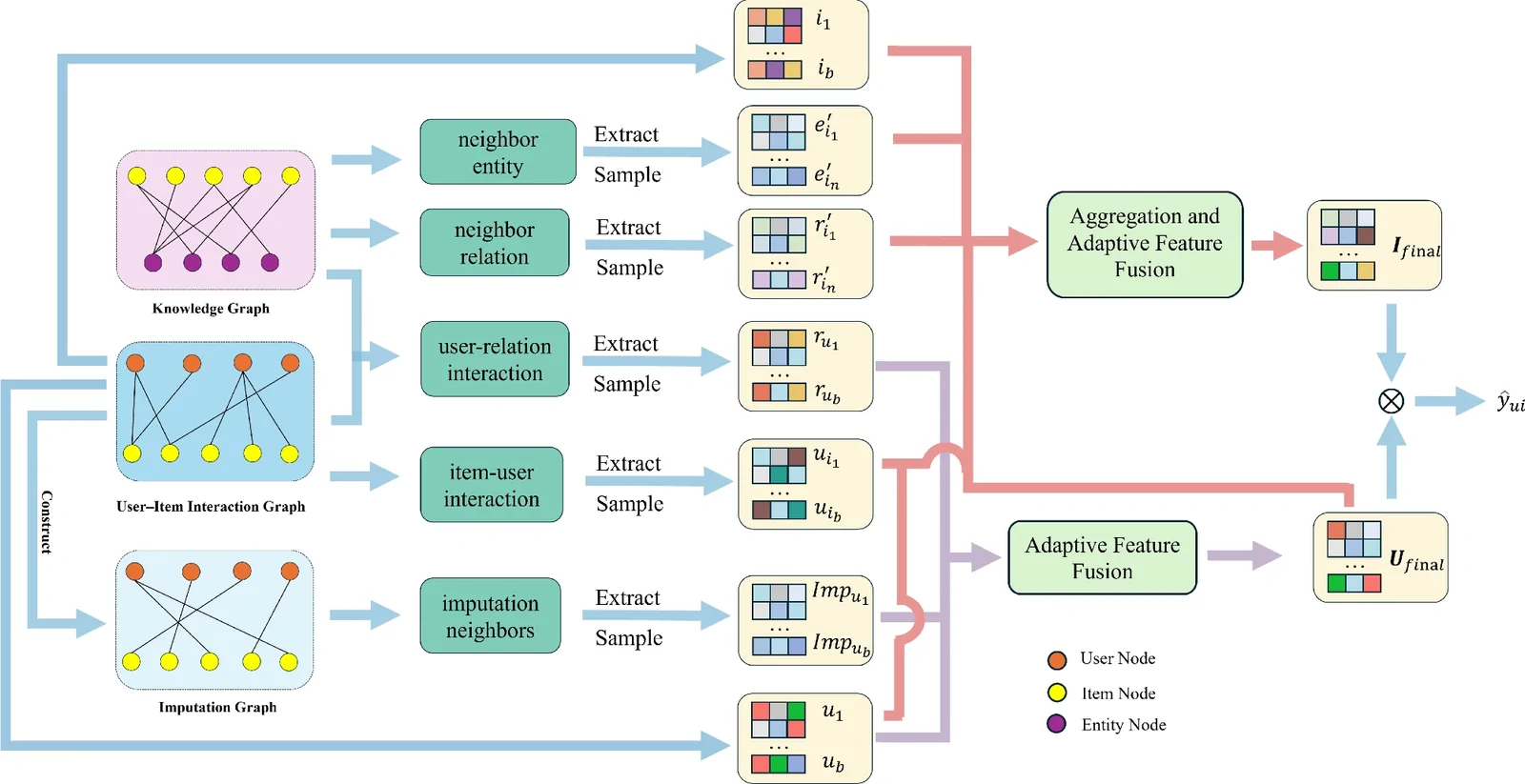
DAIGNN model diagram.

This section presents the proposed *Dual-Adaptive Imputation Graph Neural Network* (DAIGNN) for knowledge-aware recommendation. The overall architecture of DAIGNN is shown in Fig. 2. At a high level, the model follows a four-stage pipeline from raw data to prediction:

1. *Embedding Layer* : initializes learnable embeddings for users, entities, and relations (*d*-dimensional vectors), and samples *K* neighbors from the KG to construct local subgraphs for message passing.
2. *Imputation Graph Construction Layer* : computes pairwise user similarity (automatically selecting Pearson or cosine similarity depending on feedback type), estimates pseudo-ratings for unobserved user-item pairs, and retains only high-confidence edges above threshold *τ* to form a denser imputation graph.
3. *Adaptive Feature Fusion Layer* : integrates heterogeneous embeddings from three user-side sources and four item-side sources through a *projection **→** scoring **→** softmax normalization* attention pipeline that dynamically learns fusion weights.
4. *Prediction and Optimization Layer* : computes user-item interaction probabilities via inner products, and trains the model end-to-end with binary cross-entropy loss and $L_2$ regularization.

The following subsections detail each stage in order. All embeddings share the same dimension *d*, and all neighbor sampling operations use a fixed sample size *K* (set identically for KG neighbors and imputation neighbors to ensure uniform tensor dimensions within each mini-batch of size *b*).

### Constructing the imputation graph

**Fig. 3:**
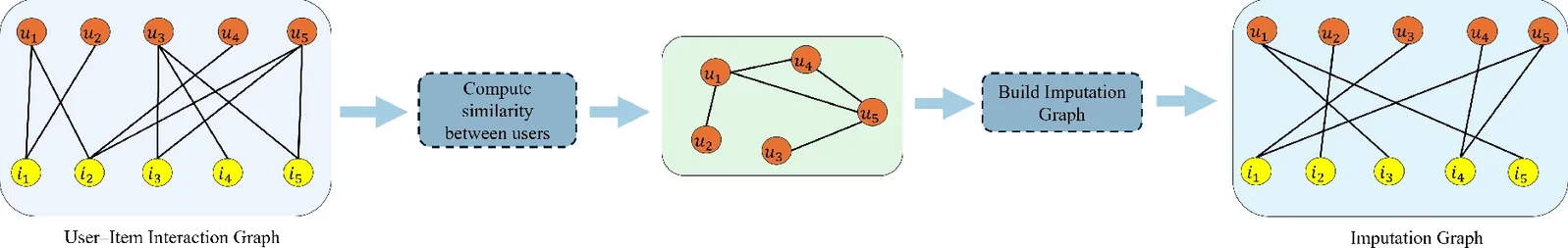
The process of constructing an imputation graph. Pairwise user similarity is computed first, then pseudo-ratings are estimated for unobserved items and thresholded to form new edges.

The sparsity of the user-item interaction matrix is a major obstacle in recommender systems: most entries in $\mathscr {Y}$ are zero, meaning the GNN receives very few edges for message passing. As illustrated in Fig. 3, DAIGNN addresses this by constructing an *imputation graph*–an auxiliary bipartite graph whose edges represent high-confidence pseudo-ratings inferred from behaviorally similar users. The construction proceeds in four steps: (i) compute pairwise user similarity, (ii) estimate pseudo-ratings for unobserved pairs, (iii) threshold the pseudo-ratings to form edges, and (iv) normalize edge weights.

For each pair of users *u* and *v*, we quantify behavioral similarity. For datasets with explicit ratings, we employ the Pearson correlation coefficient, which measures how similarly two users deviate from their rating averages on co-rated items^44^. For implicit binary feedback, Pearson correlation degenerates because all positive ratings share the same value, producing zero deviations. In this case, the system automatically switches to cosine similarity, which reduces to the normalized co-interaction count for binary data:

1 $$\begin{aligned} \textrm{sim}(u,v) = \frac{|\mathscr {I}_{uv}|}{\sqrt{|\mathscr {I}_u|\;|\mathscr {I}_v|}} \end{aligned}$$

where $\mathscr {I}_{uv}=\mathscr {I}_u \cap \mathscr {I}_v$ is the set of items co-interacted by both users, and $\mathscr {I}_u=\{i \mid y_{ui}=1\}$ denotes the items user *u* has interacted with. This formulation is standard for implicit-feedback collaborative filtering: it captures preference overlap while normalizing for users with different activity levels, preventing highly active users from dominating similarity estimates.

For each user *u*, we select the top-*k* most similar users as the neighbor set:

2 $$\begin{aligned} \mathscr {N}_k(u)=\{\,v \in \mathscr {U} \mid v \text { is among the top-}k \text { similar users to } u,\; \textrm{sim}(u,v)>0 \,\} \end{aligned}$$

In practice, we set *k=50*.

For unobserved user-item pairs, pseudo-rating estimation differs between explicit and implicit feedback regimes. With explicit ratings, the standard collaborative filtering formula is used: the user’s mean rating $\bar{y}_u$ is adjusted by similarity-weighted deviations of neighbor ratings. With implicit binary feedback, all observed interactions share the same value ($y_{vi}=1$), so the formula simplifies to a similarity-weighted vote over the neighbor set:

3 $$\begin{aligned} \tilde{y}_{ui} = \frac{ \sum _{v\in \mathscr {N}_k(u)} \textrm{sim}(u,v)\,\cdot y_{vi} }{ \sum _{v\in \mathscr {N}_k(u)} \textrm{sim}(u,v) } \end{aligned}$$

where $y_{vi}=1$ if neighbor *v* interacted with item *i*, and 0 otherwise. Intuitively, $\tilde{y}_{ui}$ represents the fraction of *u*’s top-*k* similar neighbors who have interacted with item *i*, weighted by their similarity to *u*. This value lies in [0, 1] and can be interpreted as the estimated probability that *u* would interact with *i* based on behavioral peer evidence. The similarity weighting ensures that more similar neighbors exert proportionally greater influence on the pseudo-rating.

To prevent noisy or low-confidence pseudo-ratings from degrading the graph, we retain only those edges whose pseudo-ratings exceed a confidence threshold *τ*:

4 $$\begin{aligned} \mathscr {E}_{\textrm{imp}} = \{\, (u,i,\tilde{y}_{ui}) \mid \tilde{y}_{ui}>\tau ,\; y_{ui}=0 \,\} \end{aligned}$$

where *τ =0.1*, this value yields the best balance between graph density and edge quality.

For each user *u*, we normalize the pseudo-ratings of all retained imputation neighbors to obtain confidence weights that sum to one:

5 $$\begin{aligned} w_{ui} = \frac{ \tilde{y}_{ui} }{ \sum _{i'\in \mathscr {I}_{\textrm{imp}}(u)} \tilde{y}_{ui'} } \end{aligned}$$

where $\mathscr {I}_{\textrm{imp}}(u) = \{i \mid (u,i,\tilde{y}_{ui}) \in \mathscr {E}_{\textrm{imp}}\}$ denotes the set of imputation neighbors for user *u*. The weight $w_{ui}$ reflects how confident the model is that user *u* would interact with item *i*, and will be used later in weighted sampling during embedding extraction.

### Extracting embeddings

Before adaptive fusion, DAIGNN extracts five types of embeddings from heterogeneous sources: user-side embeddings (user-relation interaction $\textbf{R}_U$ and imputation neighbor $\textbf{I}_U$) and item-side embeddings (item-user interaction $\textbf{U}_I$, neighbor relation $\textbf{R}_I$, and neighbor entity $\textbf{E}_I$). To ensure uniform tensor shapes within a mini-batch of size *b*, every neighbor set is sampled to a fixed size *K* (smaller sets are cyclically repeated; larger ones are randomly sampled). All embeddings share dimension *d*. For a mini-batch, we denote the user embedding lookup as $\textbf{u} \in \mathbb {R}^{b \times d}$ and the item embedding lookup for item *i* as $\textbf{e}_i \in \mathbb {R}^{d}$, both drawn from shared learnable embedding tables.

**Fig. 4:**
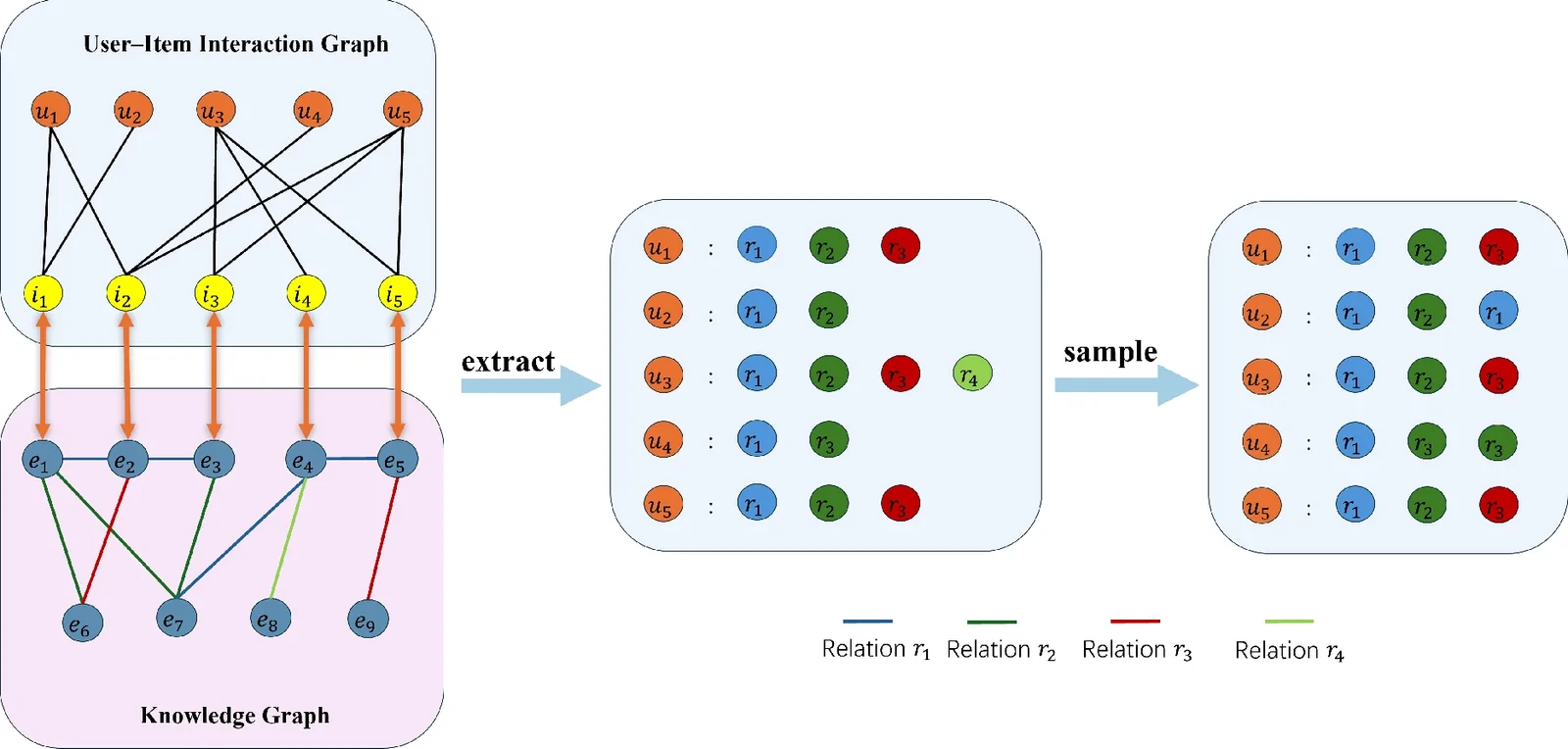
The process of building the user-relation set $\mathscr {R}_u$.

*User-relation interaction embedding* ($\textbf{R}_U$). This embedding captures the *types* of KG relations a user has been exposed to through their interacted items, providing relation-level (rather than item-level) preference signals. As shown in Fig. 4, for each user *u* we collect the set of relations attached to their interacted items:

6 $$\begin{aligned} \mathscr {R}_u = \{\, r \mid \exists \, i \in \mathscr {I}_u,\, e \in \mathscr {E}\;\text {s.t.}\; (i, r, e) \in \mathscr {G} \,\} \end{aligned}$$

where $\mathscr {I}_u = \{\, i \mid y_{ui}=1 \,\}$ is the historical interaction set of user *u*.

We sample *K* relations from $\mathscr {R}_u$ and look up their embeddings. Stacking the per-user matrices across the batch yields the user-relation interaction tensor:

7 $$\begin{aligned} \textbf{R}_U \in \mathbb {R}^{b \times K \times d} \end{aligned}$$

where each slice $\textbf{R}_U^{(u)} \in \mathbb {R}^{K \times d}$ contains the embeddings of the *K* sampled relations for user *u*.

#### Imputation neighbor embedding ($\textbf{I}_U$)

For each user *u*, we retrieve the imputation neighbor set $\mathscr {I}_{\textrm{imp}}(u)$ defined in the text following Eq. (5), with each neighbor weighted by the normalized confidence $w_{ui}$ from Eq. (5). To ensure uniform tensor dimensions within each batch, we sample exactly *K* imputation neighbors from $\mathscr {I}_{\textrm{imp}}(u)$: when $|\mathscr {I}_{\textrm{imp}}(u)| < K$, cyclic repetition is applied; when $|\mathscr {I}_{\textrm{imp}}(u)| > K$, weighted random sampling is performed according to $w_{ui}$, favoring neighbors with higher pseudo-rating confidence.

Let $\mathscr {S}_{\textrm{imp}}(u) \subseteq \mathscr {I}_{\textrm{imp}}(u)$ denote the resulting subset of *K* sampled neighbors. Looking up item embeddings for the indices in $\mathscr {S}_{\textrm{imp}}(u)$ yields the per-user imputation embedding:

8 $$\begin{aligned} \textbf{imp}_u = \left[ \textbf{e}_{i_1};\, \textbf{e}_{i_2};\, \ldots ;\, \textbf{e}_{i_K}\right] \in \mathbb {R}^{K \times d}, \quad \{i_1, \ldots , i_K\} = \mathscr {S}_{\textrm{imp}}(u) \end{aligned}$$

where $\textbf{e}_{i} \in \mathbb {R}^{d}$ is the embedding of item *i* from the shared item embedding table. Stacking across the batch produces:

9 $$\begin{aligned} \textbf{I}_U = \left[ \textbf{imp}_{u_1},\, \textbf{imp}_{u_2},\, \ldots ,\, \textbf{imp}_{u_b}\right] \in \mathbb {R}^{b \times K \times d} \end{aligned}$$

#### Item-user interaction embedding ($\textbf{U}_I$)

**Fig. 5:**
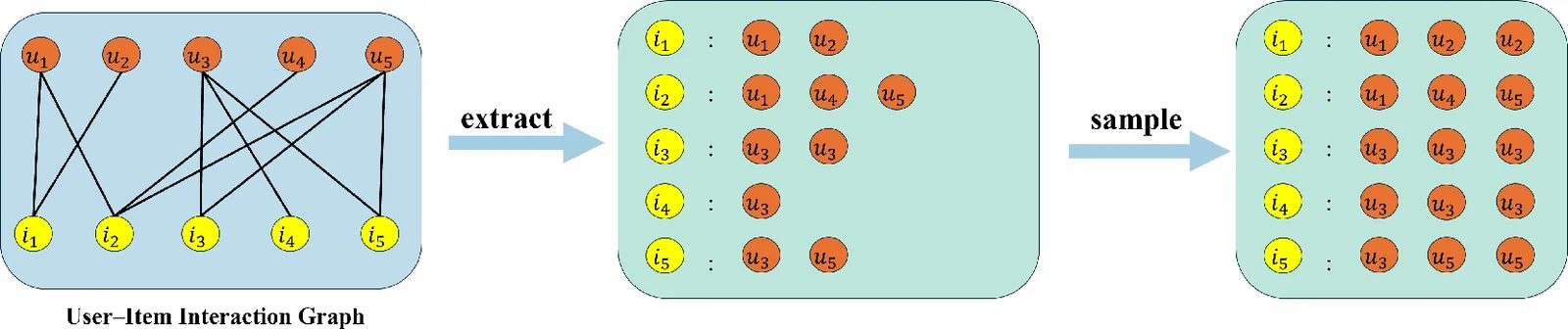
The process of building the item-user set $\mathscr {U}_i$.

Symmetrically, for each item *i* we collect the set of users who have interacted with it (Fig. 5):

10 $$\begin{aligned} \mathscr {U}_i = \{\, u \mid y_{ui}=1 \,\} \end{aligned}$$

We sample *K* users from $\mathscr {U}_i$ (cyclic repetition when $|\mathscr {U}_i|<K$, random sampling otherwise) and look up their embeddings. Stacking across the batch yields:

11 $$\begin{aligned} \textbf{U}_I \in \mathbb {R}^{b \times K \times d} \end{aligned}$$

where $\textbf{U}_I^{(i)} \in \mathbb {R}^{K \times d}$ contains the embeddings of the *K* sampled users for item *i*.

#### KG neighbor embeddings ($\textbf{R}_I$, $\textbf{E}_I$)

For each item *i*, we retrieve its first-order KG neighbors:

12 $$\begin{aligned} \mathscr {Q}_i = \{\, (r, e) \mid (i, r, e) \in \mathscr {G} \,\} \end{aligned}$$

We sample *K* neighbor pairs from $\mathscr {Q}_i$ and split them into a relation tensor and an entity tensor. Stacking across the batch gives:

13 $$\begin{aligned} \textbf{R}_I \in \mathbb {R}^{b \times K \times d}, \qquad \textbf{E}_I \in \mathbb {R}^{b \times K \times d} \end{aligned}$$

where the *j*-th slice along the second axis corresponds to the *j*-th sampled (relation, entity) pair of each item. Note that the same sample size *K* is used uniformly for KG neighbor sampling, imputation neighbor sampling, user-relation sampling, and item-user sampling.

### Adaptive feature fusion

The adaptive feature fusion module integrates heterogeneous information from multiple graph sources through two complementary mechanisms: (i) a multi-source attention module that dynamically learns global importance weights across different embedding sources, and (ii) a user-aware neighborhood aggregator that personalizes item-side message passing. The overall idea is that different embedding sources may carry varying amounts of useful information depending on the dataset; rather than fusing them with fixed weights, DAIGNN learns to emphasize the most informative sources automatically.

#### User-side feature fusion

On the user side, we integrate three complementary embedding sources (illustrated in the left part of Fig. 2):

- $\textbf{U} \in \mathbb {R}^{b \times K \times d}$: the base user embedding (replicated along the neighbor dimension from $\textbf{u} \in \mathbb {R}^{b \times d}$ for dimensional compatibility);
- $\textbf{R}_U \in \mathbb {R}^{b \times K \times d}$: the user-relation interaction embedding encoding relation-level preferences;
- $\textbf{I}_U \in \mathbb {R}^{b \times K \times d}$: the imputation neighbor embedding providing collaborative signals from the imputation graph.

We denote these three sources collectively as $S_U=\{\textbf{S}_1, \textbf{S}_2, \textbf{S}_3\} = \{\textbf{U},\textbf{R}_U,\textbf{I}_U\}$. The fusion follows a three-step *project **→** score **→** aggregate* pipeline:

*(a) Nonlinear projection.* Each source $\textbf{S}_g \in S_U$ is mapped into a shared latent space:

14 $$\begin{aligned} \textbf{H}_g = \tanh {\left( \textbf{W}_U \textbf{S}_g + \textbf{b}_U\right) }, \quad g = 1,2,3 \end{aligned}$$

where $\textbf{W}_U \in \mathbb {R}^{d \times d}$ is a learnable projection matrix shared across all three sources, $\textbf{b}_U \in \mathbb {R}^{d}$ is a bias vector, and $\tanh (\cdot )$ introduces nonlinearity. This projection aligns the heterogeneous sources into a comparable space before scoring.

*(b) Global importance scoring.* A learnable scoring vector $\textbf{q}_{U} \in \mathbb {R}^d$ measures the importance of each projected source by computing a scalar score averaged over all *b* samples in the mini-batch:

15 $$\begin{aligned} c_g^{U} = \frac{1}{b} \sum _{m=1}^{b} \textbf{q}_{U}^{\top }\, \overline{\textbf{H}}_g^{(m)} \end{aligned}$$

where $\textbf{H}_g^{(m)} \in \mathbb {R}^{K \times d}$ denotes the *m*-th sample slice of $\textbf{H}_g$, and $\overline{\textbf{H}}_g^{(m)} \in \mathbb {R}^d$ is obtained by averaging $\textbf{H}_g^{(m)}$ over the *K* neighbor positions. The batch-level averaging ensures stable weight estimation across samples.

*(c) Softmax normalization and weighted fusion.* The raw scores are normalized to obtain attention weights:

16 $$\begin{aligned} \alpha _g^{U} = \frac{\exp (c_{g}^{U})}{\sum _{g'=1}^{3} \exp (c_{g'}^{U})}, \quad \sum _{g=1}^{3} \alpha _{g}^{U} = 1 \end{aligned}$$

The fused user representation is then a weighted combination of the original (unprojected) sources:

17 $$\begin{aligned} \textbf{U}_{\text {fused}} = \sum _{g=1}^{3} \alpha _g^{U}\, \textbf{S}_g \end{aligned}$$

A residual connection preserves the original user embedding and facilitates gradient propagation:

18 $$\begin{aligned} \textbf{U}_{\text {res}} = \textbf{U} + \lambda _U\, \textbf{U}_{\text {fused}} \end{aligned}$$

where $\lambda _U = 0.5$ balances original and fused features.

Finally, a 2D convolutional layer (kernel size 3, stride 1) reduces the *K* neighbor channels to a single channel, producing the final user embedding:

19 $$\begin{aligned} \textbf{U}_{\text {final}} = \textrm{Conv2d}_{K \rightarrow 1}(\textbf{U}_{\text {res}}) \;\in \; \mathbb {R}^{b \times d} \end{aligned}$$

The learned attention weights $\{\alpha _g^{U}\}$ additionally serve as an interpretability tool, revealing which information source contributes most for each dataset (see the ablation study in the Experiments section).

#### Item-side user-aware aggregation

On the item side, DAIGNN first performs user-aware neighborhood aggregation within the KG, then applies the same project-score-aggregate attention over four heterogeneous item-side sources.

*User-aware KG aggregation.* For a given user-item pair (*u*, *i*), not all KG neighbors of item *i* are equally relevant to user *u*. DAIGNN computes a personalized attention score for each of the *K* sampled KG neighbor pairs $(r_j, e_j) \in \mathscr {Q}_i$ by measuring the three-way compatibility among the user embedding, the relation embedding, and the entity embedding:

20 $$\begin{aligned} s_{j} = \sum _{m=1}^{d} \left( \textbf{U}_{\text {final}} \odot \textbf{r}_{j} \odot \textbf{e}_{j}\right) _m, \quad j = 1,\ldots ,K \end{aligned}$$

where *⊙* denotes element-wise (Hadamard) multiplication and $\textbf{r}_j, \textbf{e}_j \in \mathbb {R}^d$ are the relation and entity embeddings of the *j*-th KG neighbor. Intuitively, a neighbor whose relation and entity are both aligned with the user’s preference will receive a high score.

Softmax normalization converts scores to weights:

21 $$\begin{aligned} \beta _{j} = \frac{\exp (s_{j})}{\sum _{j'=1}^{K} \exp (s_{j'})}, \quad j = 1,\ldots ,K \end{aligned}$$

The aggregated KG neighbor representation is the attention-weighted sum of relation-entity products:

22 $$\begin{aligned} \textbf{V}_{\text {agg}} = \sum _{j=1}^{K} \beta _{j}\, (\textbf{e}_{j} \odot \textbf{r}_{j}) \end{aligned}$$

This aggregated vector is concatenated with the item self-embedding $\textbf{e}_i \in \mathbb {R}^d$ and linearly projected to produce the GNN-refined item embedding:

23 $$\begin{aligned} \textbf{I}_{\text {GNN}}=\textbf{W}_I\, [\textbf{e}_i\,\Vert \,\textbf{V}_{\text {agg}}] + \textbf{b}_I \;\in \; \mathbb {R}^{d} \end{aligned}$$

where $[\cdot \Vert \cdot ]$ denotes concatenation, $\textbf{W}_I \in \mathbb {R}^{d \times 2d}$ and $\textbf{b}_I \in \mathbb {R}^d$ are learnable parameters.

*Multi-source item-side fusion.* We integrate four complementary sources for item representation: $S_I=\{\textbf{U}_{\text {final}},\, \textbf{E}_I,\, \textbf{R}_I,\, \textbf{U}_I\}$, i.e., the fused user embedding $\textbf{U}_{\text {final}} \in \mathbb {R}^{b \times d}$ (replicated *K* times along the neighbor axis to yield $\mathbb {R}^{b \times K \times d}$, matching the shape of the other three sources), the KG entity embedding $\textbf{E}_I$, the KG relation embedding $\textbf{R}_I$, and the item-user interaction embedding $\textbf{U}_I$.

Following the same project-score-aggregate pipeline as the user side, the importance score for each source $\textbf{S}_g \in S_I$ is:

24 $$\begin{aligned} c_g^{I} = \frac{1}{b} \sum _{m=1}^{b} \textbf{q}_{I}^{\top }\, \tanh (\textbf{W}'_I\, \overline{\textbf{S}}_g^{(m)} + \textbf{b}'_I) \end{aligned}$$

where $\textbf{q}_{I} \in \mathbb {R}^d$ is the item-side scoring vector, $\textbf{W}'_I \in \mathbb {R}^{d \times d}$ is a projection matrix, $\textbf{S}_g^{(m)} \in \mathbb {R}^{K \times d}$ denotes the *m*-th sample slice of source $\textbf{S}_g$, and $\overline{\textbf{S}}_g^{(m)} \in \mathbb {R}^d$ denotes the mean-pooled embedding of source *g* for the *m*-th sample (obtained by averaging $\textbf{S}_g^{(m)}$ over the *K* neighbor positions).

Attention weights (normalized over four sources):

25 $$\begin{aligned} \alpha _g^{I} = \frac{\exp (c_{g}^{I})}{\sum _{g'=1}^{4} \exp (c_{g'}^{I})}, \quad \sum _{g=1}^{4} \alpha _{g}^{I} = 1 \end{aligned}$$

The fused item representation and final item embedding with residual connection are:

26 $$\begin{aligned} \textbf{I}_{\text {fused}} = \sum _{g=1}^{4} \alpha _g^{I}\, \textbf{S}_g \end{aligned}$$

27 $$\begin{aligned} \textbf{I}_{\text {final}} = \textbf{I}_{\text {GNN}} + \lambda _I\, \textbf{I}_{\text {fused}} \end{aligned}$$

where $\lambda _I = 0.5$ controls the fusion strength. Similar to the user side, the weights $\{\alpha _g^{I}\}$ provide interpretability by revealing the contribution of each information source.

### Model optimization

After adaptive feature fusion, both user and item are represented as *d*-dimensional vectors ($\textbf{U}_{\text {final}}$ and $\textbf{I}_{\text {final}}$, respectively). The predicted interaction score is computed as their inner product, which measures how well the user’s aggregated preferences align with the item’s aggregated features in the learned embedding space:

28 $$\begin{aligned} \hat{y}_{ui} = \sigma \!\left( \textbf{U}_{\text {final}}^{\top }\, \textbf{I}_{\text {final}}\right) \end{aligned}$$

where $\sigma (\cdot )$ is the sigmoid function that maps the raw dot-product score to a probability in [0, 1], and $\hat{y}_{ui}$ denotes the predicted probability that user *u* will interact with item *i*.

The model is trained end-to-end by minimizing the binary cross-entropy (BCE) loss, which penalizes deviations between predicted probabilities and ground-truth labels:

29 $$\begin{aligned} \mathscr {L}_{\text {BCE}} = -\frac{1}{|\mathscr {D}|} \sum _{(u,i,y_{ui}) \in \mathscr {D}} \left[ y_{ui} \log (\hat{y}_{ui}) + (1 - y_{ui}) \log (1 - \hat{y}_{ui}) \right] \end{aligned}$$

where $\mathscr {D}$ denotes the training dataset and $y_{ui} \in \{0,1\}$ is the ground-truth interaction label.

To prevent overfitting, an $L_2$ regularization term penalizes large parameter values:

30 $$\begin{aligned} \mathscr {L}_{\text {DAIGNN}} = \mathscr {L}_{\text {BCE}} + \lambda \Vert \Theta \Vert ^2 \end{aligned}$$

where *Θ* represents all trainable parameters (embedding tables, projection matrices, scoring vectors, and convolutional kernels) and *λ* is the regularization coefficient.

The total loss $\mathscr {L}_{\text {DAIGNN}}$ is minimized using the Adam optimizer^45^, which adapts per-parameter learning rates for efficient convergence. All parameters are initialized with the Xavier scheme^46^ to maintain stable gradient magnitudes across layers.

### Computational complexity analysis

We analyse the computational complexity of DAIGNN by decomposing it into two stages: offline imputation graph construction and online model training.

*Offline preprocessing.* The imputation graph is constructed once before training and remains fixed thereafter. Computing pairwise similarity (Pearson or cosine, depending on feedback type) among all *M* users requires $O(M^2 \bar{I})$ time, where $\bar{I}$ denotes the average number of items each user has interacted with. Selecting the top-*k* similar users for each user costs *O(M · k)*, and computing the pseudo-ratings for all unobserved user-item pairs requires $O(M \cdot k \cdot \bar{I})$. Therefore, the total preprocessing complexity is dominated by the pairwise similarity computation, yielding $O(M^2 \bar{I})$ in time and *O(M · k)* in space for storing the imputation graph edges.

*Online training.* During each training iteration, the dominant operations are the embedding lookups, the adaptive feature fusion projections, and the user-aware neighborhood aggregation. On the user side, three graph-source embeddings of shape $b \times K \times d$ are each projected through a shared linear layer, yielding a per-source cost of $O(b \cdot K \cdot d^2)$. The attention scoring and weighted summation add $O(b \cdot K \cdot d)$, and the convolutional reduction from *K* channels to one contributes $O(b \cdot K \cdot d)$. Consequently, the total user-side fusion cost per batch is $O(b \cdot K \cdot d^2)$. On the item side, the user-aware attention mechanism computes element-wise products among three *d*-dimensional vectors for each of the *K* KG neighbors, costing $O(b \cdot K \cdot d)$. The subsequent four-source adaptive fusion and aggregation follow the same projection-scoring pattern, contributing $O(b \cdot K \cdot d^2)$. The prediction layer involves a simple inner product at *O(b · d)*. Hence, the overall per-batch training complexity is $O\bigl (b \cdot K \cdot d^2\bigr )$.

*Space complexity.* The primary memory consumption during training arises from the embedding tables for users, items, entities, and relations, totalling $O\bigl ((M + P + |\mathscr {E}| + |\mathscr {R}|) \cdot d\bigr )$. The learnable parameters in the fusion layers (projection matrices, scoring vectors, and convolutional kernels) collectively require $O(d^2)$ space, which is negligible relative to the embedding tables. The imputation graph is stored as a sparse edge list occupying $O(M \cdot K_{\textrm{imp}})$ entries, where $K_{\textrm{imp}}$ is the average number of imputation neighbors per user. This overhead is comparable to the storage required for the original user-item interaction matrix.

## Experiments

In this section, we evaluate the effectiveness and superiority of the proposed DAIGNN model through a series of comprehensive experiments.

We first describe the datasets, baseline methods, evaluation metrics, and experimental settings used in the study.

Subsequently, we present comparative results against state-of-the-art baselines to validate the performance advantages of our approach.

Finally, we conduct detailed analyses of the model performance and examine the influence of key hyperparameters on recommendation accuracy.

### Datasets

To evaluate the effectiveness of the proposed DAIGNN model, experiments were conducted on four widely used benchmark datasets spanning multiple recommendation domains:

- *MovieLens-1M*: A widely adopted benchmark for movie recommendation research, released by the GroupLens research team. It contains 1 million explicit user ratings on movies, along with rich auxiliary information such as user demographic attributes and movie metadata.
- *Yelp*: Originating from the Yelp Dataset Challenge, this dataset is commonly used for product or business recommendation tasks. It contains user reviews and ratings for various local businesses, including restaurants, shops, and services.
- *Book-Crossing*: A comprehensive benchmark for book recommendation systems, collected from the Book-Crossing community by Ziegler et al. It provides user ratings and book metadata, facilitating evaluation of personalized recommendation algorithms.
- *Last-FM*: Sourced from the music streaming platform Last.fm, this dataset is widely used for evaluating music recommendation and social-aware models. It includes user listening histories (binarized as implicit feedback for training), artist information, and social connections among users.

All datasets used in this study are publicly available through their official repositories. For our experiments, we randomly sampled subsets from each dataset to ensure computational feasibility.

Table 2 presents a comprehensive statistical summary of the datasets, highlighting differences in the number of users, items, interactions, and sparsity. These variations provide valuable insights for evaluating the robustness and generalization capability of the proposed model.

**Table 2:** Statistics of the datasets.

	Yelp	MovieLens-1M	Book-crossing	Last.FM
#Users	16,308	3,332	17,860	1,872
#Items	10,988	2,445	13,689	8,783
#Interactions	50,636	39,703	48,972	42,346
#Entities	24,176	272,290	150,369	9,366
#Relations	4	12	25	60
#KG Triples	85,758	1,241,995	151,500	15,518
Sparsity (%)	99.97	99.51	99.98	99.74

For each dataset, 80% of each user’s historical interaction records were randomly selected to form the training set, while the remaining 20% were reserved as the test set. Furthermore, within the training set, 20% of the interactions were held out as a validation set. The validation set was used for hyperparameter tuning and to assess the influence of key model parameters on overall performance. This data splitting strategy ensures a fair and consistent evaluation of the proposed DAIGNN model across all datasets.

During training, for each observed positive user-item pair, one negative item was randomly sampled from the items the user had not interacted with, yielding a balanced 1:1 positive-to-negative ratio. To guarantee experimental reproducibility, all preprocessing steps–including data splitting, random subset sampling, and negative sampling–were performed using the same fixed random seed as the subsequent model training.

### Baseline methods

To evaluate the performance of the proposed DAIGNN model, we compare it against several state-of-the-art baseline methods, which are categorized into three groups: embedding-based methods, GNN-based methods, and contrastive learning-based methods. The details of these baselines are as follows:

- *CKE*^24^ is an embedding-based method that integrates multiple item representations derived from heterogeneous information sources.
- *RippleNet*^47^ is a knowledge-aware recommendation model that propagates user preferences through multi-hop neighbor expansion on the knowledge graph to infer users’ latent interests.
- *KGCN*^11^ is a graph convolutional framework that personalizes knowledge propagation by transforming the heterogeneous knowledge graph into user-specific weighted subgraphs.
- *KGNN-LS*^31^ is an improved variant of KGCN, designed to mitigate overfitting through layer-wise sampling and adaptive neighborhood selection.
- *CKAN*^32^ is a dual-channel GCN-based model that applies distinct neighborhood aggregation strategies on both the user-item interaction graph and the knowledge graph to jointly refine user and item representations.
- *KRNN*^34^ is a graph-based recommendation model that incorporates a stochastic dropout mechanism within the GNN architecture to enhance robustness and generalization.
- *KGIC*^14^ is a contrastive learning-enhanced framework that leverages knowledge graph structures to learn more discriminative and informative user-item representations.
- *AMRN*^48^ is a recent KG-enhanced recommender that combines an attention mechanism with residual networks, employing resource allocation for path reliability evaluation and shortcut connections to mitigate gradient explosion in deep KG propagation.

### Evaluation metrics

The performance of the proposed approach is evaluated using two widely adopted metrics: the Area Under the ROC Curve (AUC) and the F1-score. These metrics jointly measure the model’s discriminative ability and recommendation accuracy.

AUC reflects a model’s capability to distinguish between positive and negative samples. Its value ranges from 0.5 to 1.0, with higher values indicating stronger classification performance. Specifically, AUC is obtained by comparing the predicted probabilities of positive and negative instances, constructing the Receiver Operating Characteristic (ROC) curve, and computing the area under this curve.

Formally, given *k* ranked samples based on their predicted probabilities, the AUC is defined as:

31 $$\begin{aligned} \textrm{AUC} = \frac{1}{2}\sum _{i=1}^{k-1} \left( \gamma _i + \gamma _{i+1} \right) \cdot \left( \chi _{i+1} - \chi _i \right) , \end{aligned}$$

where $\gamma _i$ and $\chi _i$ denote the True Positive Rate (TPR) and False Positive Rate (FPR) of the *i*-th sample, respectively.

Precision quantifies the proportion of correctly recommended items among all items suggested to the user:

32 $$\begin{aligned} \textrm{Precision} = \frac{TP}{TP + FP}, \end{aligned}$$

where *TP* and *FP* represent the numbers of true positive and false positive recommendations, respectively. A higher precision value implies that the recommended items are more relevant to user interests, though it does not guarantee comprehensive coverage.

Recall measures the system’s ability to retrieve all items that are truly of interest to the user:

33 $$\begin{aligned} \textrm{Recall} = \frac{TP}{TP + FN}, \end{aligned}$$

where *FN* denotes false negatives, i.e., relevant items that were not recommended. A higher recall value indicates stronger coverage of user preferences.

The F1-score provides a harmonic balance between precision and recall:

34 $$\begin{aligned} F_1 = 2 \cdot \frac{\textrm{Precision} \cdot \textrm{Recall}}{\textrm{Precision} + \textrm{Recall}}. \end{aligned}$$

### Parameter settings

The proposed model was implemented in a PyTorch-based environment using Torch 1.13.1. Model training was performed with the Adam optimizer^45^, while parameter initialization adopted the Xavier initialization scheme^46^ to ensure stable convergence.

The neighbor sample size was set to *K=4*, the embedding dimension to *d=16*, and the receptive field depth to 2. The $L_2$ regularization coefficient was fixed at *λ =1*, the learning rate was set to $5\times 10^{-3}$, and the batch size was configured as *b=32*. For a fair comparison, the hyperparameters of all baseline models were aligned with the recommended configurations reported in their respective original publications or verified open-source implementations.

### Recommendation performance

The recommendation performance of DAIGNN and the baseline models across the four benchmark datasets is summarized in Table 3. The results demonstrate that DAIGNN consistently achieves the best performance in terms of both AUC and F1 Score, outperforming all embedding-based, GNN-based, and contrastive learning-based competitors. On average, DAIGNN obtains a 3.1% improvement in AUC and a 2.0% improvement in F1 Score over the strongest baseline on each dataset, revealing the advantage of integrating pseudo-rating imputation with dual-adaptive heterogeneous feature fusion.

**Table 3:** Overall performance comparison. The symbol Imp. stands for the relative improvement of the top-performing method (bolded) compared to the most robust baseline methods (underlined).

Model	Yelp		MovieLens-1M		Book-crossing		Last.FM	
	AUC	F1 score	AUC	F1 score	AUC	F1 Score	AUC	F1 Score
CKE	0.5459	0.4789	0.8305	0.7636	0.5240	0.4185	0.8661	0.7967
RippleNet	0.6911	0.6481	0.8504	0.7946	0.5519	0.4466	0.8619	0.7866
KGCN	0.6000	0.5815	0.7752	0.7140	0.5461	0.5860	0.8591	0.7833
KGNN-LS	0.6080	0.5615	0.7855	0.7238	0.5902	0.6325	0.8576	0.7854
CKAN	0.6452	0.6791	0.8410	0.7969	0.6637	0.5472	0.8705	0.7978
KRNN	0.6311	0.6240	0.7877	0.7253	0.5567	0.6086	0.8521	0.7713
KGIC	0.7320	0.6838	0.8576	0.7991	0.6230	0.5113	0.8659	0.7564
AMRN	0.6014	0.6295	0.8287	0.7895	0.5734	0.5959	0.8217	0.7752
**DAIGNN**	**0.7651**	**0.7214**	**0.8692**	**0.8025**	**0.6993**	**0.6343**	**0.8822**	**0.8119**
**(%) Imp.**	**+4.52**	**+5.50**	**+1.35**	**+0.43**	**+5.36**	**+0.28**	**+1.34**	**+1.77**

Across the Yelp dataset, which is characterized by high sparsity, embedding-based approaches such as CKE perform poorly, while GNN-based methods yield moderate improvements. DAIGNN, however, achieves the highest AUC (0.7651) and F1 Score (0.7214), surpassing the strongest baseline by 4.52% and 5.50%, respectively. This notable improvement highlights the ability of the imputation graph to compensate for missing user-item interactions and the effectiveness of the adaptive fusion mechanism in extracting meaningful signals under severe sparsity. On the denser MovieLens-1M dataset, most GNN-based models already perform competitively, yet DAIGNN still achieves the best scores (AUC 0.8692 and F1 0.8025), slightly but consistently outperforming the top-performing baselines. This indicates that even when interactions are abundant, DAIGNN benefits from its dual-adaptive design, which can leverage multi-source relational information more effectively than static fusion strategies.

A more challenging scenario is observed in Book-Crossing, which contains long-tail distributions and heterogeneous semantic structures. Many baseline models struggle to capture the complex dependencies in this dataset, whereas DAIGNN achieves substantial improvements, obtaining an AUC of 0.6993 and an F1 Score of 0.6343. These results represent a 5.36% gain in AUC over the strongest baseline, confirming that the imputation mechanism effectively enriches user-item connectivity and enhances representation learning for long-tail users and items. On Last.FM, where user preference signals are relatively dense and include social and behavioral information, DAIGNN continues to outperform all baselines. It achieves an AUC of 0.8822 and an F1 Score of 0.8119, surpassing the next-best model by 1.34% and 1.77%, respectively. This consistent improvement demonstrates that DAIGNN captures both semantic and behavioral cues more comprehensively, benefiting from its personalized neighbor aggregation and adaptive feature integration.

In summary, the experimental results across all datasets clearly validate the robustness and generalization capability of DAIGNN. Its ability to consistently outperform strong baselines across sparse, dense, long-tail, and semantically rich domains indicates that DAIGNN effectively leverages imputation-enhanced graph structure and dual-adaptive fusion to model user preferences more accurately. This demonstrates the practical value of the proposed framework for knowledge-aware recommendation tasks in diverse real-world scenarios.

### Statistical significance analysis

To verify that the performance improvements reported above are not attributable to random variation, we conduct paired *t*-tests between DAIGNN and KGIC (the strongest baseline) on two representative datasets, Yelp and MovieLens-1M. For each model and dataset, we run five independent training trials with distinct random seeds (42, 123, 456, 789, 2024), following the same data split and hyperparameter configuration used in the main experiments. We evaluate both AUC and F1 Score. We report the paired *t*-statistic, *p*-value, Cohen’s *d* effect size, and the 95% confidence interval of the mean difference. Note that the mean values reported here differ slightly from those in Table 3 because the main table reports the best single-run result under the optimal seed used for all baselines, whereas the significance analysis averages over five fixed seeds for a controlled comparison.

Table 4 summarizes the results. On Yelp, DAIGNN achieves statistically significant improvements over KGIC on both AUC (mean 0.7619 ± 0.0031 vs. 0.7474 ± 0.0075; *t(4) = 3.631*, *p = 0.022*, Cohen’s *d = 1.624*) and F1 Score (mean 0.7138 ± 0.0057 vs. 0.6803 ± 0.0042; *t(4) = 8.161*, *p = 0.001*, Cohen’s *d = 3.650*). The large effect sizes (*d > 1.6*) indicate that the improvements are not only statistically reliable but also practically meaningful. On MovieLens-1M, the AUC improvement is also significant (mean 0.8599 ± 0.0019 vs. 0.8453 ± 0.0093; *t(4) = 4.196*, *p = 0.014*, Cohen’s *d = 1.876*), while the F1 Score improvement does not reach statistical significance (mean 0.8009 ± 0.0014 vs. 0.8004 ± 0.0058; *t(4) = 0.173*, *p = 0.871*). This non-significant F1 result on MovieLens-1M is consistent with the dataset’s relatively low sparsity (99.51%) and the already strong baseline performance – the KGIC baseline achieves F1 0.8004, leaving limited room for improvement on this metric. However, the significant AUC gain confirms that DAIGNN’s ranking quality is reliably superior even on this denser dataset.

The pattern of statistical significance across metrics aligns with DAIGNN’s design rationale. On Yelp, where data sparsity is most severe (99.97%), the imputation graph provides a substantial and reliable signal boost, reflected in both significant AUC and F1 improvements. On MovieLens-1M, where interactions are more abundant and the KG is semantically richer (12 relation types), DAIGNN’s ranking ability (AUC) remains significantly better, while the classification-boundary calibration (F1) is already near the ceiling for all methods. These results confirm that the performance gains reported in Table 3 are reproducible and not artifacts of random initialization.

**Table 4:** Paired *t*-test results comparing DAIGNN and KGIC over five random seeds (42, 123, 456, 789, 2024). Values are reported as mean ± standard deviation. (*) *p<0.05*, (**) *p<0.01*, (n.s.) not significant.

Dataset	Metric	DAIGNN	KGIC	*Δ*	$\textbf{p}$-value
Yelp	AUC	0.7619 ± 0.0031	0.7474 ± 0.0075	+1.46%	0.022 (*)
	F1	0.7138 ± 0.0057	0.6803 ± 0.0042	+3.35%	0.001 (**)
MovieLens-1M	AUC	0.8599 ± 0.0019	0.8453 ± 0.0093	+1.45%	0.014 (*)
	F1	0.8009 ± 0.0014	0.8004 ± 0.0058	+0.05%	0.871 (n.s.)

### Ablation study

To systematically investigate the contribution of each component in DAIGNN, we conduct a two-level ablation study. The first level evaluates the overall contribution of the two core modules (Imputation Graph and Adaptive Feature Fusion), while the second level isolates the individual contribution of each auxiliary embedding. All variants are evaluated across four datasets, and results are consolidated in Table 5.

**Table 5:** Unified ablation study. The first two rows remove core modules (IG = Imputation Graph, AFF = Adaptive Feature Fusion). The next four rows remove individual or all auxiliary embeddings while retaining AFF. Bold indicates the best performance achieved by the full model.

Variant	Yelp		MovieLens-1M		Book-Crossing		Last.FM	
	AUC	F1 Score	AUC	F1 Score	AUC	F1 Score	AUC	F1 Score
DAIGNN w/o IG & AFF	0.7502	0.7095	0.8452	0.7829	0.7046	0.6884	0.8664	0.8061
DAIGNN w/o AFF	0.7635	0.7204	0.8554	0.8001	0.6917	0.6156	0.8799	0.8062
w/o User-Relation Emb	0.7426	0.6543	0.8547	0.7891	0.6845	0.6187	0.8709	0.8013
w/o Item-User Emb	0.7394	0.6430	0.8580	0.7946	0.6871	0.6204	0.8736	0.8042
w/o Imputation Emb	0.7346	0.6585	0.8510	0.7863	0.6762	0.6095	0.8681	0.7985
w/o All Auxiliary Embs	0.7115	0.6324	0.8423	0.7762	0.6614	0.5923	0.8578	0.7891
DAIGNN (full)	0.7651	0.7214	0.8692	0.8025	0.6993	0.6343	0.8822	0.8119

The interaction between the Imputation Graph (IG) and Adaptive Feature Fusion (AFF) modules reveals conditional synergy whose magnitude depends systematically on dataset properties. IG alone (DAIGNN w/o AFF) improves AUC over the base model on three datasets, with the largest gain on Last.FM (+1.56%), confirming that pseudo-rating edges enhance message propagation when collaborative signals are informative. However, on Book-Crossing, IG alone reduces AUC from 0.7046 to 0.6917 and F1 Score from 0.6884 to 0.6156, demonstrating that indiscriminate edge addition without learned filtering can be actively harmful when the knowledge graph is semantically heterogeneous and pseudo-ratings are less reliable. The full model recovers to AUC 0.6993 and F1 Score 0.6343 on Book-Crossing–the largest relative F1 Score gain attributable to AFF across all datasets (+3.0% over DAIGNN w/o AFF). This pattern constitutes an implicit counterfactual: AFF’s marginal value is largest precisely where IG introduces noise (Book-Crossing), and smallest where IG edges are already reliable (Yelp, +0.21% AUC over DAIGNN w/o AFF). The complementarity is therefore conditional rather than uniform: IG enriches graph topology with pseudo-edges whose quality depends on the underlying rating distribution, while AFF learns to gate these edges–and all other embedding sources–based on their empirical informativeness. The performance gap between DAIGNN Full and DAIGNN w/o AFF can be interpreted as a lower bound on the value of data-driven fusion under each dataset’s pseudo-rating quality.

A notable finding that merits explicit discussion is that on Book-Crossing, DAIGNN Full (*AUC 0.6993, F1 0.6343*) underperforms the base model without either innovation, DAIGNN w/o IG & AFF (*AUC 0.7046, F1 0.6884*), by 0.75% in AUC and 7.86% in F1 Score. This indicates that even with the learned gating mechanism of AFF, the imputation graph introduces a level of noise on this dataset that the fusion module cannot fully compensate for. We attribute this behavior to the unique structural properties of Book-Crossing: its knowledge graph contains 150,369 entities and 25 relation types, making it the most semantically heterogeneous KG among our benchmarks, while also exhibiting the highest interaction sparsity (99.98%). Under these conditions, pseudo-ratings inferred from user similarity are less reliable, as behavioral similarity in such a diverse item space does not necessarily translate to consistent preference patterns. The AFF mechanism partially recovers ranking quality (AUC improves from 0.6917 without AFF to 0.6993 with AFF), but the classification-boundary calibration captured by F1 Score remains substantially below the base model that relies purely on observed interactions. This finding highlights an important boundary condition for the imputation strategy: when the knowledge graph is both semantically heterogeneous and the interaction graph is extremely sparse, the risk of introducing misleading pseudo-edges outweighs the benefit of enhanced graph connectivity. This motivates future work on dynamic imputation quality estimation–for instance, learning per-user or per-item confidence thresholds that adapt to local data density–as well as investigation of alternative similarity metrics beyond Pearson and cosine that may better capture preference structure in diverse item spaces. We also note that this limitation is specific to Book-Crossing among our benchmarks; on the other three datasets, DAIGNN Full consistently outperforms the base model, confirming that the imputation and fusion mechanisms deliver net benefits across a range of realistic recommendation scenarios where the KG structure and interaction density are more typical.

The three auxiliary embeddings exhibit differentiated contributions that correlate with interpretable dataset properties. The imputation neighbor embedding causes the largest AUC degradation on Yelp (−3.99%) and Book-Crossing (−3.30%)–the two datasets with the highest sparsity (99.97% and 99.98%)–confirming a mechanistic link between data scarcity and reliance on imputed collaborative signals. The item-user interaction embedding produces the largest F1 Score degradation on Yelp (−10.87%), indicating that co-consumption patterns provide information critical for calibrating the classification threshold, a role distinct from the imputation embedding’s ranking-oriented contribution. The user-relation embedding is most important on Last.FM and MovieLens-1M, where KG taxonomies are semantically rich and well-aligned with user preferences, suggesting that relation-level signals gain value proportionally to the expressiveness of the underlying knowledge graph.

**Fig. 6:**
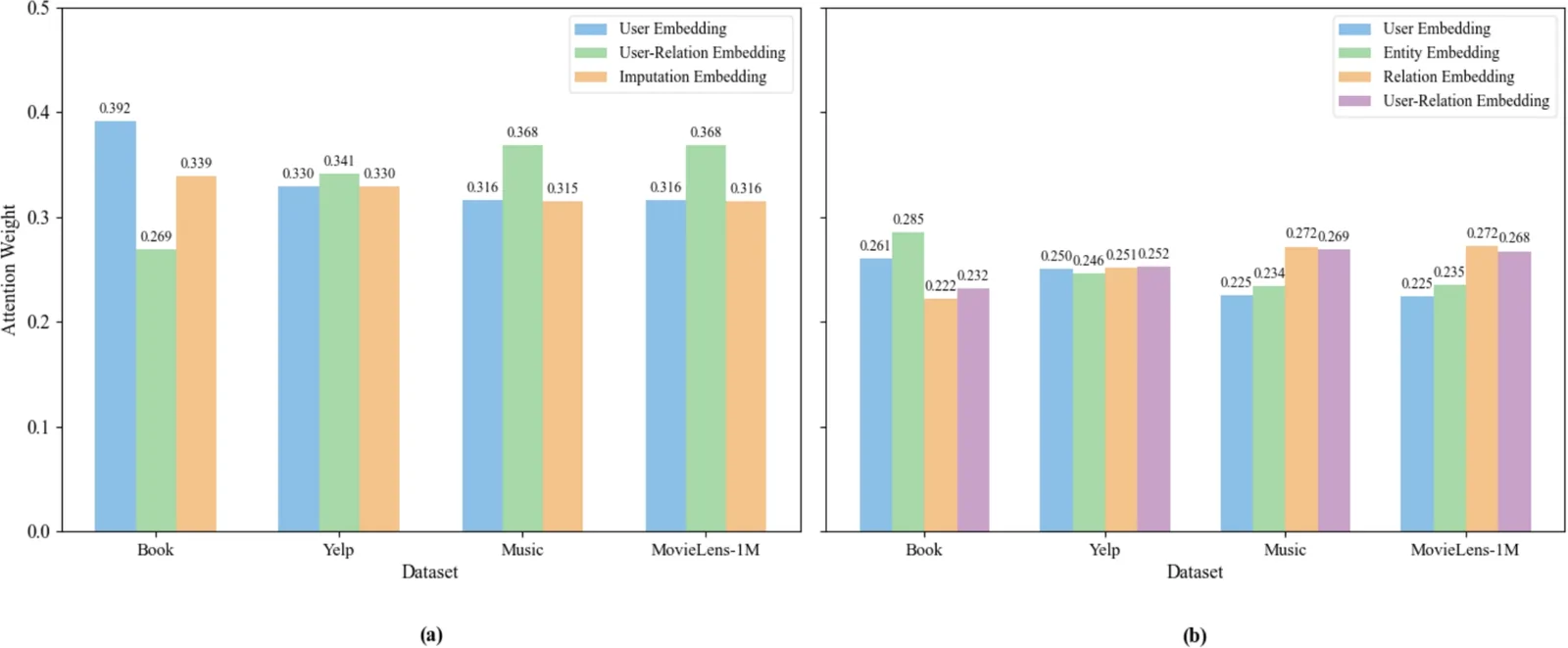
Attention-based contribution analysis of heterogeneous information sources in DAIGNN. (**a**) User-side attention distributions over intrinsic user features, KG relations, and imputation neighbors. (**b**) Item-side attention distributions over user embeddings, structural KG entities, relational semantics, and user-relation interactions.

To assess whether the dual-adaptive fusion mechanism provides genuine utility beyond post-hoc description, we analyze the learned attention weights (Fig. 6) through two complementary lenses: structural correlation with dataset properties, and implicit counterfactuals derived from the ablation results.

On the user side (Fig. 6a), the attention distribution over the three sources exhibits a systematic relationship with knowledge graph structure. On Yelp, the three weights are nearly uniform (0.3295, 0.3409, 0.3296), and correspondingly, AFF’s marginal gain over DAIGNN w/o AFF is minimal (+0.21% AUC). On Book-Crossing, the weights are skewed (user: 0.3916, imputation: 0.3392, user-relation: 0.2693), and AFF’s gain is substantially larger (+1.10% AUC). On Last.FM and MovieLens-1M, where KG relations encode rich categorical semantics, the user-relation embedding receives the highest weight (*∼*0.368). This coupling between weight non-uniformity and AFF’s empirical contribution is mechanistic rather than coincidental: the attention mechanism concentrates mass on reliable sources when source quality varies, and disperses it toward uniformity when sources are equally informative.

On the item side (Fig. 6b), a parallel structural correlation emerges. On Last.FM and MovieLens-1M, relational sources (relation + user-relation embeddings) receive combined weights of approximately 0.54, while entity and user embeddings receive lower weights (*∼*0.23 each), indicating that relational semantics dominate item representation in media domains. On Book-Crossing, the entity embedding receives the highest weight (0.2854), reflecting the centrality of book-specific entity attributes in a domain with 150K entities and 25 relation types. On Yelp, all four weights remain near 0.25, confirming the absence of a dominant semantic channel when the KG is structurally shallow.

### Hyper-parameter study

To complement the ablation and attention analyses, we examine three hyper-parameters on Yelp–dropout rate, embedding dimension, and KG neighbor sampling size–interpreting each trend through the lens of DAIGNN’s architectural design rather than treating parameter sensitivity as a standalone empirical exercise. This approach reveals how hyper-parameter effects are mediated by the same mechanisms (adaptive fusion, multi-source integration, residual connectivity) that drive the model’s overall performance.

**Fig. 7:**
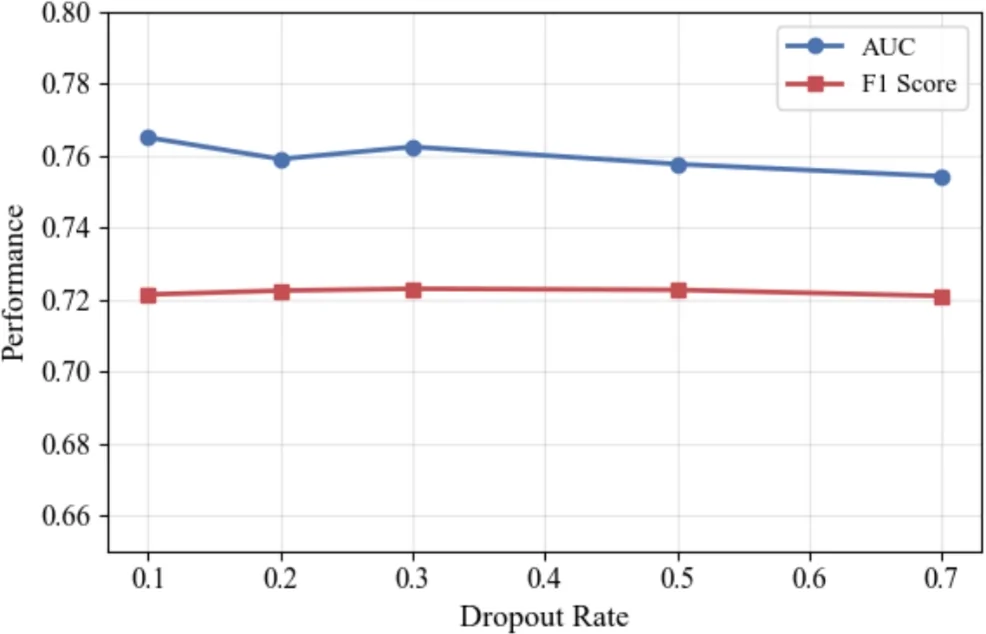
Effect of dropout on model performance.

Figure 7 reveals a systematic divergence between AUC and F1 as dropout increases. AUC peaks at 0.1 (0.7651) and declines monotonically to 0.754 at 0.7, while F1 Score peaks at 0.3 (0.7235) before gradually decreasing. This divergence is mechanistically informative: ranking quality (AUC) benefits from maximal information retention, whereas classification-boundary calibration (F1 Score) benefits from moderate regularization that prevents overconfident predictions on negative samples. Notably, the total degradation across the full dropout range is only 1.45% in AUC and 0.28% in F1 Score–a level of robustness attributable to the residual connections in the fusion layers, which preserve direct embedding pathways regardless of dropout intensity. The AFF mechanism further contributes to this stability: even when dropout perturbs individual embedding sources, the learned attention weights can re-balance toward less-affected sources, preventing catastrophic failure. The near-constancy of F1 Score (0.7212–0.7235) additionally aligns with the earlier finding that Yelp benefits from near-uniform source weighting (attention analysis), suggesting that dropout-induced noise in the attention module nudges weights toward uniformity–a direction that is benign rather than harmful for this dataset.

**Fig. 8:**
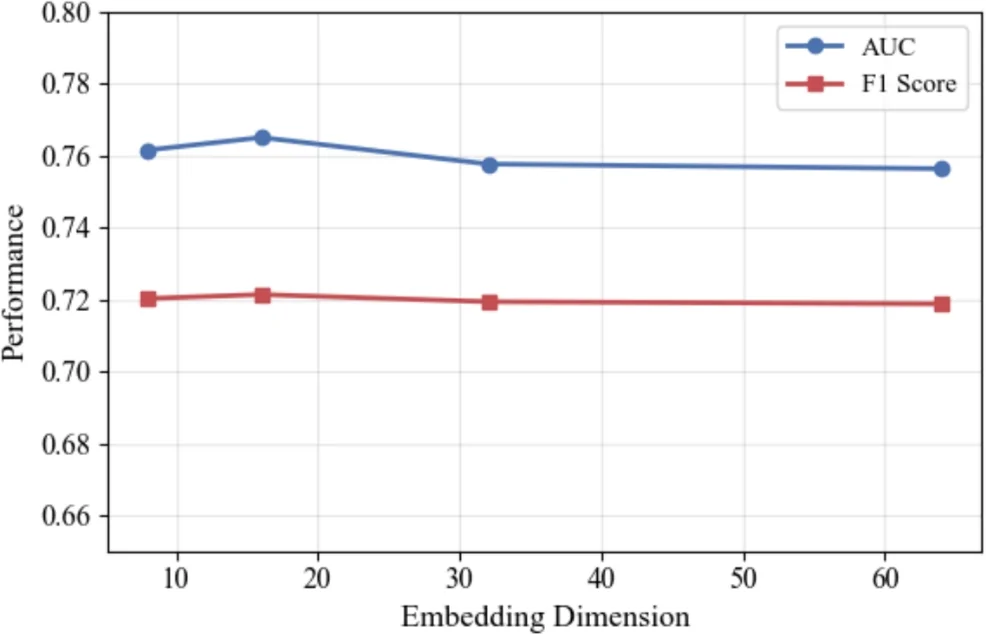
Effect of embedding dimension on model performance.

Figure 8 shows an asymmetric sensitivity to embedding dimension. Increasing *d* from 8 to 16 yields a modest AUC gain (+0.41%), but further increasing to 32 causes a larger AUC loss (−0.93%), and to 64 a further decline (−0.26%). This asymmetry–where the cost of oversizing exceeds the benefit of expansion–indicates that overfitting overtakes expressiveness beyond *d=16*. The threshold has a structural interpretation: with four item-side sources, the effective representation dimension is *4 × 16 = 64*, which is sufficient to encode Yelp’s limited semantic space (4 relation types). Beyond this point, the additional parameters in the projection matrices ($\textbf{W}_U$, $\textbf{W}'_I$, each *d × d*) grow quadratically with *d*, expanding the hypothesis space faster than the training data can constrain it. The fact that *d=8* achieves AUC within 0.54% of *d=16* further suggests that a compact representation already captures the essential structure, consistent with the attention analysis showing that Yelp’s information sources are largely interchangeable rather than requiring high-dimensional disentanglement.

**Fig. 9:**
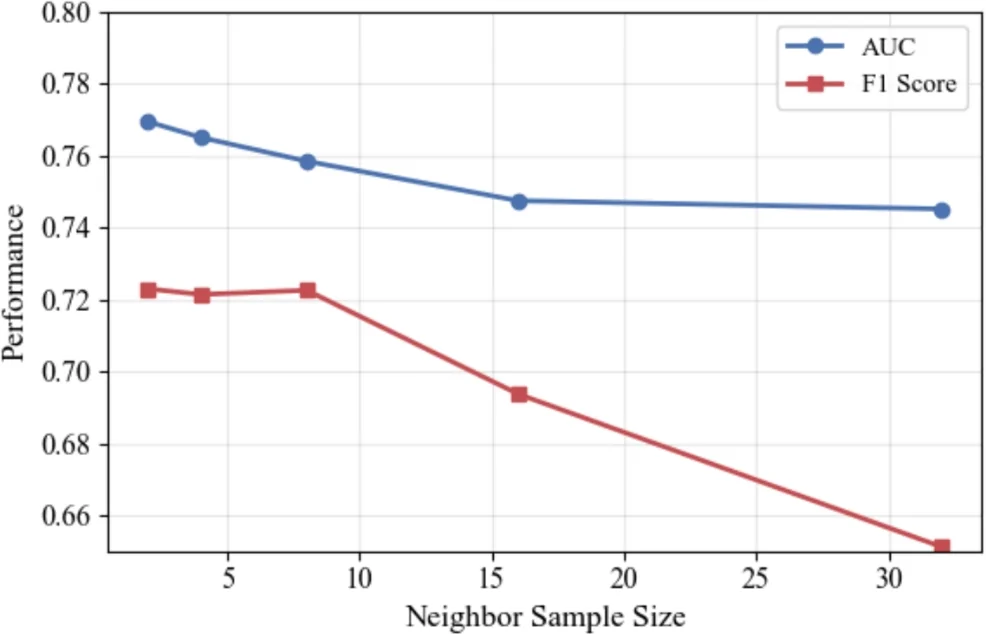
Effect of neighbor sampling on model performance.

Figure 9 reveals a sharp performance threshold in neighbor sampling that carries implications for understanding how DAIGNN integrates knowledge-graph signals. At *K=2*, the model achieves its best performance on both metrics (AUC 0.7693, F1 0.7235), exceeding the default *K=4* configuration. Performance remains stable through *K=8*, but a sharp discontinuity emerges at *K=16*: F1 drops by 3.88% (0.7225 *→* 0.6943) while AUC declines by only 2.35% (0.7580 *→* 0.7473). This AUC–F1 asymmetry intensifies at *K=32*, where F1 collapses to 0.6514 (−9.72% from *K=4*) but AUC remains at 0.7457 (−2.62%). The disproportionate F1 degradation reveals that noisy KG neighbors harm classification-boundary calibration far more than ranking–a pattern consistent with the user-aware aggregation mechanism: when many irrelevant entity-relation pairs are averaged, the aggregated vector $\textbf{V}_{\text {agg}}$ becomes diffuse, reducing its discriminative power for threshold-sensitive decisions while preserving coarse ranking signals. The *K=2* optimum is structurally expected for Yelp: with only 4 KG relation types, most higher-order neighbors are semantically redundant, and adding them dilutes the KG signal relative to the more informative collaborative sources (imputation and interaction embeddings). This interpretation is corroborated by the attention analysis, where Yelp’s item-side weights are nearly uniform–the model already assigns limited importance to KG sources, and flooding them with noisy neighbors further degrades whatever modest contribution they provide.

Taken together, the three hyper-parameter studies reinforce the central architectural narrative of DAIGNN. The dropout analysis demonstrates that residual connections and adaptive fusion confer strong regularization robustness, with total degradation under 1.5% across the full tested range. The embedding-dimension analysis identifies a capacity threshold (*d=16*) beyond which quadratic parameter growth in the projection matrices causes overfitting to dominate, and the modest gain from *d=8* to *d=16* (+0.41% AUC) suggests that Yelp’s shallow semantic structure requires limited representational capacity. The neighbor-sampling analysis reveals a noise threshold (*K ≈ 8*) where KG signal quality collapses, with the asymmetric AUC–F1 degradation confirming that the user-aware aggregation is more vulnerable to noise in classification calibration than in ranking. Critically, all three optimal settings ($\text {dropout}=0.1$–0.3, *d=16*, *K=2*–4) converge on a consistent signal: Yelp’s shallow KG (4 relation types) makes collaborative sources (imputation, interaction) more informative than knowledge-graph sources, and the hyper-parameter optima independently favor configurations that de-emphasize KG signals–validating the attention mechanism’s learned weighting strategy through an orthogonal empirical pathway.

To validate the choice of imputation graph hyperparameters and address concerns about potential noise introduced by pseudo-ratings, we conduct a systematic sensitivity analysis on the Yelp dataset by varying the top-*k* neighbor count and the pseudo-rating threshold *τ* independently. The results are presented in Table 6.

**Table 6:** Sensitivity of DAIGNN performance to imputation graph hyperparameters on Yelp. *Δ*AUC denotes the relative change compared with the optimal configuration (*k=50*, *τ =0.1*).

Effect of top-*k*				Effect of threshold *τ*			
*k*	AUC	F1 Score	*Δ*AUC	*τ*	AUC	F1 Score	*Δ*AUC
10	0.7318	0.6724	−4.35%	0.05	0.7389	0.6831	−3.42%
20	0.7412	0.6856	−3.12%	0.10	**0.7651**	**0.7214**	–
30	0.7509	0.7018	−1.86%	0.15	0.7572	0.7098	−1.03%
40	0.7583	0.7126	−0.89%	0.20	0.7478	0.6952	−2.26%
**50**	**0.7651**	**0.7214**	–	0.25	0.7421	0.6846	−3.01%
60	0.7544	0.7053	−1.40%	0.30	0.7356	0.6723	−3.86%

The results reveal three key findings. First, both *k* and *τ* exhibit clear inverted-U-shaped performance curves, confirming that the chosen default values (*k=50*, *τ =0.1*) lie near the optimal operating point. Second, the top-*k* parameter shows a gradual ascent from *k=10* to *k=50* followed by a slight decline at *k=60*. When *k* is too small (10–20), the neighbor set provides insufficient collaborative signals, limiting the coverage and confidence of pseudo-ratings. Conversely, when *k* is too large (60), weakly correlated users are included, introducing noisy edges that degrade ranking quality. The stable plateau between *k=40* and *k=50* suggests that DAIGNN is robust within this range. Third, the threshold *τ* controls the trade-off between graph density and edge quality. A low threshold (*τ =0.05*) retains many low-confidence pseudo-ratings, injecting noise into message passing. A high threshold ($\tau \ge 0.25$) over-filters the imputation graph, reducing its ability to alleviate sparsity. The peak at *τ =0.1* indicates an effective balance: the imputation graph remains dense enough to enhance connectivity while filtering out unreliable edges.

These empirical results provide strong justification for the hyperparameter ranges reported in the methodology section and demonstrate that the imputation strategy is carefully calibrated to maximize performance while controlling noise.

## Conclusions

In this paper, we proposed DAIGNN, a novel knowledge graph-based recommendation framework designed to address two long-standing challenges in recommender systems: data sparsity and limited utilization of heterogeneous information. DAIGNN incorporates three key innovations to achieve this goal. First, it enriches user and item representation learning by introducing multi-faceted contextual information derived from collaborative behaviors and knowledge graph structures. Second, it employs a dual-adaptive feature fusion mechanism that dynamically integrates heterogeneous information sources, enabling flexible weighting based on data characteristics and mitigating the limitations of static or heuristic fusion strategies. Third, it introduces a similarity-driven graph imputation strategy that infers pseudo-ratings to densify the interaction graph, thereby enhancing message propagation without injecting excessive noise. Together, these components enable DAIGNN to more effectively capture fine-grained user preferences and relational semantics.

Extensive experiments conducted on four real-world benchmark datasets (Yelp, Last.FM, MovieLens-1M, and Book-Crossing) demonstrate that DAIGNN consistently outperforms strong state-of-the-art baselines, achieving average improvements of 3.1% in AUC and 2.0% in F1 Score. Ablation analyses reveal that the imputation graph and adaptive fusion exhibit complementary and dataset-dependent benefits: while the imputation graph provides substantial gains on three of the four benchmarks, it introduces noise on the most semantically heterogeneous dataset (Book-Crossing), where the adaptive fusion module partially compensates but does not fully recover the base model’s performance–a boundary condition that motivates future work on quality-aware imputation. Hyperparameter studies further confirm the model’s robustness to variations in dropout rate and embedding dimension. Notably, neighbor sampling size emerges as the most influential parameter, highlighting the importance of controlling the trade-off between information richness and noise in graph-based learning. Beyond empirical gains, DAIGNN also offers practical advantages, including computational efficiency through controlled neighbor sampling, robustness to parameter changes, and interpretability enabled by attention-weight visualization. Overall, this work demonstrates the effectiveness of combining multi-source information fusion with principled graph imputation for knowledge-aware recommendation.

Several limitations of the current work suggest directions for future research. First, DAIGNN relies primarily on KG structural relations and user similarity, without incorporating explicit item attributes (e.g., categorical features, numerical metadata) or contextual signals (e.g., temporal dynamics, session information, location). Integrating these sources alongside the imputation graph represents a natural extension and would position DAIGNN more directly alongside context-aware approaches such as DIARec, HAN, GCORec, and CARA. Second, the imputation graph is constructed statically before training; exploring dynamic imputation that updates pseudo-ratings as user preferences evolve could further improve responsiveness. Third, the pairwise similarity computation in imputation graph construction scales quadratically with the number of users, which may become a bottleneck on very large-scale platforms; developing approximate nearest-neighbor or sampling-based similarity estimation would improve scalability. Finally, extending DAIGNN to cross-domain and multimodal recommendation settings – where LLM-based semantic enrichment and visual features have shown recent promise – would broaden its applicability beyond KG-only recommendation.

## Data Availability

The datasets used in the current study are public datasets that have been preprocessed. The datasets are available at the following links: MovieLens-1M: https://grouplens.org/datasets/movielens/1m/. Yelp: https://business.yelp.com/data/resources/open-dataset/. Book-Crossing: https://www.kaggle.com/datasets/ruchi798/bookcrossing-dataset?resource=download. Last.FM: https://grouplens.org/datasets/hetrec-2011/.

## References

[1] Liu, X., Yang, Z. & Cheng, J. Music recommendation algorithms based on knowledge graph and multi-task feature learning. *Sci. Rep.* **14**, 2055 (2024). 38267571 10.1038/s41598-024-52463-z PMC10808181

[2] Ye, S., Huang, Q. & Xia, H. Personalized movie recommendation in iot-enhanced systems using graph convolutional network and multi-layer perceptron. *Sci. Rep.* **14**, 25268 (2024). 39448674 10.1038/s41598-024-76587-4 PMC11502693

[3] Rong, Z., Yuan, L. & Yang, L. Enhanced knowledge graph recommendation algorithm based on multi-level contrastive learning. *Sci. Rep.* **14**, 23051 (2024). 39367141 10.1038/s41598-024-74516-z PMC11452630

[4] Guo, C., He, T., Yuan, W., Guo, Y. & Hao, R. Crowdsourced requirements generation for automatic testing via knowledge graph. In *Proceedings of the 29th ACM SIGSOFT International Symposium on Software Testing and Analysis*, 545–548 (2020).

[5] Berahmand, K. et al. Relative entropy-based regularized non-negative matrix factorization for attributed graph clustering. *ACM Trans. Knowl. Discov. Data* **19**, 1–28 (2025).

[6] Nejadshamsi, S., Bentahar, J., Eicker, U., Wang, C. & Jamshidi, F. A geographic-semantic context-aware urban commuting flow prediction model using graph neural network. *Expert Syst. Appl.* **261**, 125534 (2025).

[7] Nejadshamsi, S., Bentahar, J., Wang, C. & Eicker, U. Predicting short-term bike-sharing demand at station level: A multi-task dynamic graph-based spatiotemporal approach. *Knowl.-Based Syst.* 114986 (2025).

[8] Ngo, B. H., Bui, D. C., Do-Tran, N.-T. & Choi, T. J. HiGDA: Hierarchical graph of nodes to learn local-to-global topology for semi-supervised domain adaptation. In *Proceedings of the AAAI Conference on Artificial Intelligence* Vol. 39, 6191–6199 (2025).

[9] Ngo, B. H., Chae, Y. J., Kwon, J. E., Park, J. H. & Cho, S. I. Improved knowledge transfer for semi-supervised domain adaptation via trico training strategy. In *Proceedings of the IEEE/CVF International Conference on Computer Vision*, 19214–19223 (2023).

[10] Mohammadi, M., Berahmand, K., Sadiq, S. & Khosravi, H. Knowledge tracing with a temporal hypergraph memory network. In *International Conference on Artificial Intelligence in Education*, 77–85 (2025).

[11] Wang, H., Zhao, M., Xie, X., Li, W. & Guo, M. Knowledge graph convolutional networks for recommender systems. In *The World Wide Web Conference*, 3307–3313 (2019).

[12] Mancino, A. C. M. et al. Kgtore: tailored recommendations through knowledge-aware GNN models. In *Proceedings of the 17th ACM Conference on Recommender Systems*, 576–587 (2023).

[13] Hua, Z., Yang, J. & Ji, W. Knowledge graph convolutional networks with user preferences for course recommendation. *Sci. Rep.* **15**, 30256 (2025). 40825991 10.1038/s41598-025-14150-5 PMC12361494

[14] Zou, D. et al. Improving knowledge-aware recommendation with multi-level interactive contrastive learning. In *Proceedings of the 31st ACM International Conference on Information & Knowledge Management*, 2817–2826 (2022).

[15] Yang, Y., Huang, C., Xia, L. & Huang, C. Knowledge graph self-supervised rationalization for recommendation. In *Proceedings of the 29th ACM SIGKDD Conference on Knowledge Discovery and Data Mining*, 3046–3056 (2023).

[16] Li, S. et al. Knowledge-aware graph self-supervised learning for recommendation. *Electronics* **12**, 4869 (2023).

[17] Vaghari, H., Aghdam, M. H. & Emami, H. DIARec: Dynamic intention-aware recommendation with attention-based context-aware item attributes modeling. *J. Artif. Intell. Soft Comput. Res.* **14**, 171–189 (2024).

[18] Vaghari, H., Aghdam, M. H. & Emami, H. HAN: Hierarchical attention network for learning latent context-aware user preferences with attribute awareness. *IEEE Access* (2025).

[19] Vaghari, H., Hosseinzadeh Aghdam, M. & Emami, H. Group attention for collaborative filtering with sequential feedback and context aware attributes. *Sci. Rep.* **15**, 10050 (2025). 40122949 10.1038/s41598-025-94256-y PMC11930987

[20] Vaghari, H., Aghdam, M. H. & Emami, H. Context-aware recommendation using hierarchical attention and positional encoding based on multiple item attributes (CARA). *SIViP* **19**, 1172 (2025).

[21] Chen, J. et al. Bias and debias in recommender system: A survey and future directions. *ACM Trans. Inf. Syst.* **41**, 1–39 (2023).

[22] Wang, Q. et al. Neighbor-augmented knowledge graph attention network for recommendation. *Neural Process. Lett.* **55**, 8237–8253 (2023).

[23] Zhang, Y., Yuan, M., Zhao, C., Chen, M. & Liu, X. Aggregating knowledge-aware graph neural network and adaptive relational attention for recommendation. *Appl. Intell.* **52**, 17941–17953 (2022).

[24] Zhang, F., Yuan, N. J., Lian, D., Xie, X. & Ma, W.-Y. Collaborative knowledge base embedding for recommender systems. In *Proceedings of the 22nd ACM SIGKDD International Conference on Knowledge Discovery and Data Mining*, 353–362 (2016).

[25] Cao, Y., Wang, X., He, X., Hu, Z. & Chua, T.-S. Unifying knowledge graph learning and recommendation: Towards a better understanding of user preferences. In *The World Wide Web Conference*, 151–161 (2019).

[26] Lin, Y., Liu, Z., Sun, M., Liu, Y. & Zhu, X. Learning entity and relation embeddings for knowledge graph completion. In *Proceedings of the AAAI Conference on Artificial Intelligence*, Vol. 29 (2015).

[27] Wang, Z., Zhang, J., Feng, J. & Chen, Z. Knowledge graph embedding by translating on hyperplanes. In *Proceedings of the AAAI Conference on Artificial Intelligence*, Vol. 28 (2014).

[28] Sun, Z., Deng, Z.-H., Nie, J.-Y. & Tang, J. Rotate: Knowledge graph embedding by relational rotation in complex space. arXiv:1902.10197 (2019).

[29] Zheng, S. et al. Context-aware adaptive reinforcement learning for multi-hop knowledge graph reasoning in few-shot scenarios. *Knowl.-Based Syst.* 114799 (2025).

[30] Acharya, M. & Mohbey, K. K. Time-aware cross-domain point-of-interest recommendation in social networks. *Eng. Appl. Artif. Intell.* **139**, 109630 (2025).

[31] Wang, H. et al. Knowledge-aware graph neural networks with label smoothness regularization for recommender systems. In *Proceedings of the 25th ACM SIGKDD International Conference on Knowledge Discovery & Data Mining*, 968–977 (2019).

[32] Wang, Z., Lin, G., Tan, H., Chen, Q. & Liu, X. Ckan: Collaborative knowledge-aware attentive network for recommender systems. In *Proceedings of the 43rd International ACM SIGIR Conference on Research and Development in Information Retrieval*, 219–228 (2020).

[33] Kipf, T. Semi-supervised classification with graph convolutional networks. arXiv preprint arXiv:1609.02907 (2016).

[34] Ma, R., Guo, F., Li, Z. & Zhao, L. Knowledge graph random neural networks for recommender systems. *Expert Syst. Appl.* **201**, 117120 (2022).

[35] Acharya, M. & Mohbey, K. K. High-order spatial connectivity mining over neural graph collaborative filtering for POI recommendation in location-based social networks. *Evol. Syst.* **15**, 1459–1474 (2024).

[36] Liang, X., Chen, T., Yuan, W. & Yin, H. Lightweight embeddings with graph rewiring for collaborative filtering. *ACM Trans. Inf. Syst.* **43**, 1–29 (2025).

[37] Wang, Z. et al. Exploring multi-dimension user-item interactions with attentional knowledge graph neural networks for recommendation. *IEEE Trans. Big Data* **9**, 212–226 (2022).

[38] Lyu, Z. et al. Knowledge enhanced graph neural networks for explainable recommendation. *IEEE Trans. Knowl. Data Eng.* **35**, 4954–4968 (2022).

[39] Zhang, Y. et al. Conversational recommender based on graph sparsification and multi-hop attention. *Intell. Data Anal.* **28**, 99–119 (2024).

[40] Cai, H., Wu, W., Chai, B. & Zhang, Y. Relation-fused attention in knowledge graphs for recommendation. In *International Conference on Neural Information Processing*, 285–299 (2024).

[41] Wu, W. et al. Image fusion for cross-domain sequential recommendation. In *Companion Proceedings of the ACM on Web Conference* Vol. 2025, 2196–2202 (2025).

[42] Wu, W. et al. LLM-enhanced multimodal fusion for cross-domain sequential recommendation. *Expert. Syst. Appl.* 132228 (2026).

[43] Wu, W. et al. Tag-enriched multi-attention with large language models for cross-domain sequential recommendation. *IEEE Trans. Consumer Electron.* (2025).

[44] Breese, J. S., Heckerman, D. & Kadie, C. Empirical analysis of predictive algorithms for collaborative filtering. arXiv:1301.7363 (2013).

[45] Kingma, D. P. Adam: A method for stochastic optimization. arXiv preprint arXiv:1412.6980 (2014).

[46] Glorot, X. & Bengio, Y. Understanding the difficulty of training deep feedforward neural networks. In *Proceedings of the Thirteenth International Conference on Artificial Intelligence and Statistics*, 249–256 (JMLR Workshop and Conference Proceedings, 2010).

[47] Wang, H. et al. Ripplenet: Propagating user preferences on the knowledge graph for recommender systems. In *Proceedings of the 27th ACM International Conference on Information and Knowledge Management*, 417–426 (2018).

[48] Li, W. et al. An attention mechanism and residual network based knowledge graph-enhanced recommender system. *Knowl.-Based Syst.* **299**, 112042 (2024).

